# Single‐Layer Spin‐Orbit‐Torque Magnetization Switching Due to Spin Berry Curvature Generated by Minute Spontaneous Atomic Displacement in a Weyl Oxide

**DOI:** 10.1002/adma.202416091

**Published:** 2025-04-24

**Authors:** Hiroto Horiuchi, Yasufumi Araki, Yuki K. Wakabayashi, Jun'ichi Ieda, Michihiko Yamanouchi, Yukio Sato, Shingo Kaneta‐Takada, Yoshitaka Taniyasu, Hideki Yamamoto, Yoshiharu Krockenberger, Masaaki Tanaka, Shinobu Ohya

**Affiliations:** ^1^ Department of Electrical Engineering and Information Systems The University of Tokyo 7‐3‐1 Hongo Bunkyo‐ku Tokyo 113–8656 Japan; ^2^ Advanced Science Research Center Japan Atomic Energy Agency 2–4 Shirakata, Tokai‐mura Naka‐Gun Ibaraki 319–1195 Japan; ^3^ NTT Basic Research Laboratories NTT Corporation 3‐1 Morinosato Wakamiya Atsugi‐shi Kanagawa 243‐0198 Japan; ^4^ Division of Electronics for Informatics Graduate School of Information Science and Technology Hokkaido University Kita 14 Nishi 9 Sapporo‐Shi Hokkaido 060–0814 Japan; ^5^ Research and Education Institute for Semiconductors and Informatics Kumamoto University 2‐39‐1 Kurokami Chuo‐Ku Kumamoto 860–8555 Japan; ^6^ Center for Spintronics Research Network (CSRN) The University of Tokyo 7‐3‐1 Hongo Bunkyo‐ku Tokyo 113–8656 Japan; ^7^ Institute for Nano Quantum Information Electronics The University of Tokyo 4‐6‐1 Komaba Meguro‐ku Tokyo 153–8505 Japan

**Keywords:** oxide electronics, spin berry curvature, spintronics, spin‐orbitronics, Weyl ferromagnet

## Abstract

Spin Berry curvature characterizes the band topology as the spin counterpart of Berry curvature and is crucial in generating novel spintronics functionalities. By breaking the crystalline inversion symmetry, the spin Berry curvature is expected to be significantly enhanced; this enhancement will increase the intrinsic spin Hall effect in ferromagnetic materials and, thus, the spin–orbit torques (SOTs). However, this intriguing approach is not applied to devices; generally, the spin Hall effect in ferromagnet/heavy‐metal *bilayer* is used for SOT magnetization switching. Here, SOT‐induced partial magnetization switching is demonstrated in a *single* layer of a single‐crystalline Weyl oxide SrRuO_3_ (SRO) with a small current density of ≈3.1 × 10^6^ A cm^−2^. Detailed analysis of the crystal structure in the seemingly perfect periodic lattice of the SRO film reveals barely discernible oxygen octahedral rotations with angles of ≈5° near the interface with a substrate. Tight‐binding calculations indicate that a large spin Hall conductivity is induced around small gaps generated at band crossings by the synergy of inherent spin‒orbit coupling and band inversion due to the rotations, causing magnetization reversal. The results indicate that a minute atomic displacement in single‐crystal films can induce strong intrinsic SOTs that are useful for spin‐orbitronics devices.

## Graphical abstract

Single‐layer magnetization switching with a small critical current density of ≈10^6^ A cm^−2^ is demonstrated. The strong intrinsic spin Hall effect, causing the switching, is induced by the synergy of the inherent spin‐orbit coupling and the spontaneous oxygen octahedral rotation in SrRuO_3_.The findings highlight the immense potential for realizing efficient material systems for spin‐orbitronics applications by precise atom positioning.

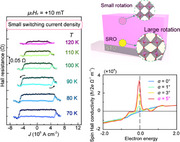

## 1 Introduction

Current‐induced spin‐orbit torque (SOT) magnetization switching has great promise for attaining high‐performance spintronics devices, such as magnetoresistive random access memory,^[^ ^1^ ^]^ nano oscillators,^[^ ^2^ ^]^ and logic devices.^[^ ^3^ ^]^ Generally, ferromagnet (FM)/heavy metal (HM) bilayer systems are used for SOT magnetization switching. In those systems, a spin current is generated from an in‐plane current in the HM layer through the spin Hall effect (SHE), exerting torques on the magnetization in the FM layer. However, when HM with strong spin‒orbit coupling (SOC) is used, the critical current density required for switching (≈10^7^ A cm^−2^) is too high for practical applications.^[^ ^4^, ^5^, ^6^ ^]^ This drawback partially arises from spin scattering at the FM/HM interface, which suppresses the SOTs. Moreover, the required switching current density scales with the FM layer thickness, while reducing the thickness increases the bit error rates. Recent breakthroughs have demonstrated magnetization switching in FM *single* layers,^[^ ^7^, ^8^, ^9^, ^10^, ^11^, ^12^, ^13^, ^14^, ^15^, ^16^, ^17^, ^18^, ^19^, ^20^, ^21^, ^22^, ^23^ ^]^ providing a promising alternative by simplifying the layer structure of the SOT devices. However, these systems require the intentional breaking of the inversion symmetry (IS) to generate SOTs, such as introducing composition gradients in the normal direction of the film.^[^ ^10^, ^12^, ^13^, ^14^, ^15^, ^17^, ^18^, ^19^, ^20^, ^21^, ^22^, ^23^ ^]^ This complexity poses significant challenges in maintaining film quality and often leads to undesired spin scattering/relaxation within the films.^[^ ^24^, ^25^ ^]^ Consequently, a novel SOT‐induced switching mechanism that operates at lower critical current densities than conventional bilayer systems while utilizing a simplified single FM layer structure is urgently needed.

**Figure 1:**
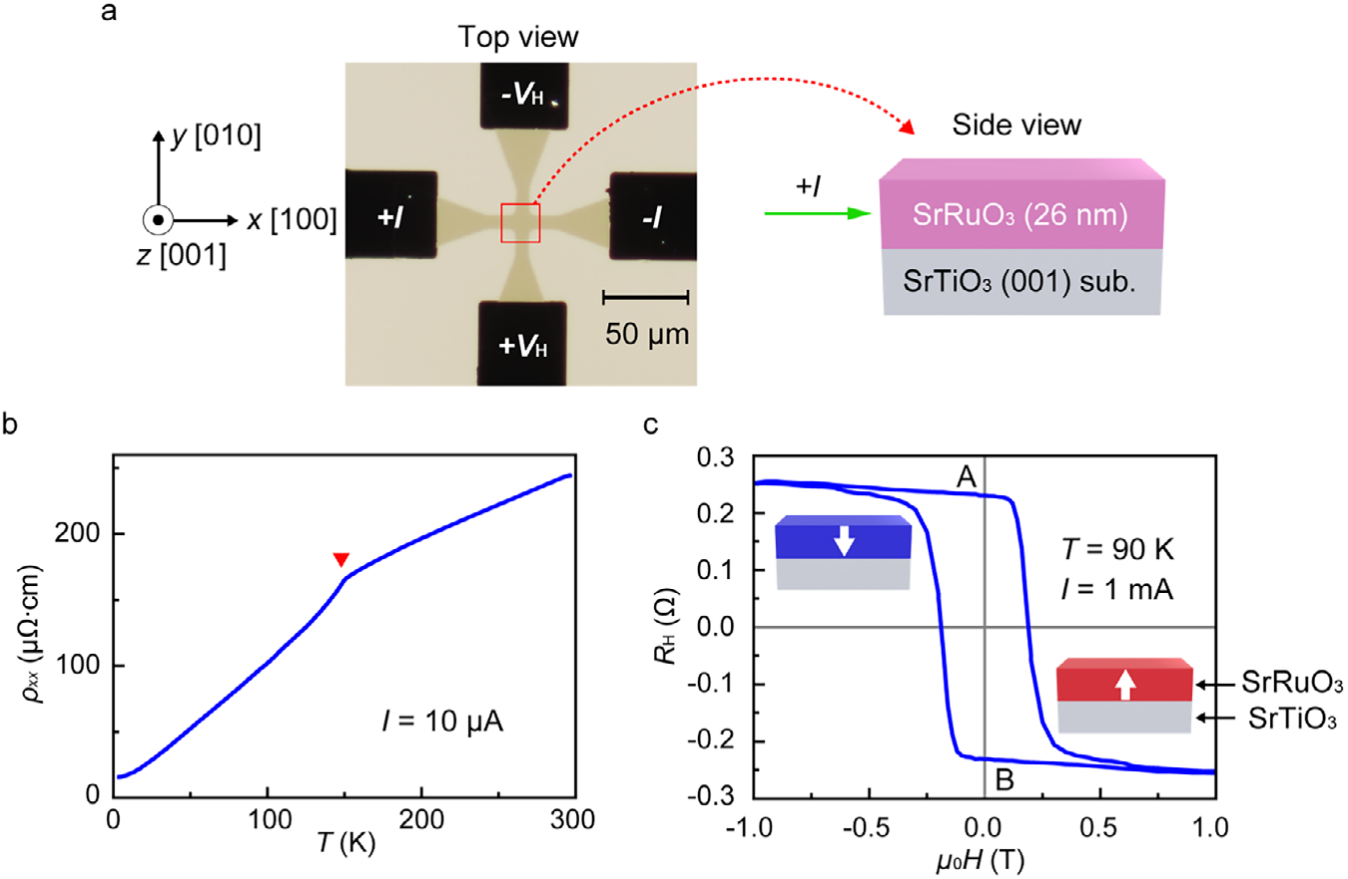
a) Optical microscope image of a crossbar device of SRO (26‐nm thick) with Ag electrodes (black square pads). A current +*I* is applied along the *x*‐axis (//[100] of the STO substrate), and the Hall voltage between the terminals +*V*_H_ and –*V*_H_ is measured to derive the Hall resistance *R*_H_ in magnetotransport measurements. b) Temperature (*T*) dependence of the resistivity (*ρ_xx_*). The red triangle indicates the Curie temperature *T*_C_ of ≈150 K. c) *R*_H_ versus external magnetic field *μ*_0_ *H* applied along the *z*‐axis at 90 K, where *R*_H_ is negatively proportional to *M_z_*. The white arrows represent the magnetization directions at positions A and B.

To address these challenges, a promising approach lies in controlling the generation and distribution of the intrinsic SHE within an FM layer while maintaining its crystal quality. The spin Berry curvature^[^ ^26^ ^]^ characterizes the band topology as the spin counterpart of the Berry curvature and determines the intrinsic SHE. Strong spin Berry curvature is predicted to appear at band crossings with band inversion in materials with strong SOC,^[^ ^27^ ^]^ generating sizable SOTs.^[^ ^28^ ^]^ The oxide Weyl ferromagnet SrRuO_3_ (SRO) has a strong SOC and emerges as a particularly intriguing candidate for this approach. It exhibits both ferromagnetism and linear band crossings in the bulk state with spatial IS.^[^ ^29^, ^30^, ^31^ ^]^ If this spatial IS can be broken while maintaining the crystal quality of SRO, a great enhancement of SOTs is expected, which will enable efficient *single‐layer* magnetization switching. Previous studies have reported that intentionally introduced magnetic domain walls break the IS of SRO, inducing efficient domain‐wall motion.^[^ ^32^, ^33^ ^]^ Here, we demonstrate current‐induced SOT partial magnetization switching in an epitaxial *single* layer of perpendicularly magnetized oxide Weyl ferromagnet SRO grown on SrTiO_3_ (STO) (001) (**Figure** **1**a). We obtain a small critical current density of ≈3.1 × 10^6^ A cm^−2^ for switching the magnetization states, one order of magnitude smaller than that required for conventional FM/HM bilayer systems^[^ ^4^, ^5^, ^6^ ^]^ and other single‐layer systems with composition gradients.^[^ ^12^, ^14^, ^17^, ^19^, ^23^ ^]^ Notably, ≈8% of the magnetization of the 26 nm‐thick SRO film is stably reversed by an in‐plane current. Our analysis using high‐angle annular dark field scanning transmission electron microscopy (HAADF‐STEM) reveals that the SRO film is a seemingly defect‐free single crystal; however, when we closely examine the lattice structure via annular bright‐field scanning transmission electron microscopy (ABF‐STEM), we find spontaneous oxygen octahedral rotations with the displacement of oxygen atoms of ≈0.01 nm near the SRO/STO interface. Our theoretical tight‐binding calculations reveal that the oxygen octahedral rotations cause band inversion around the small gaps generated at band crossings due to the broken sublattice symmetry. Consequently, the synergy between the originally existing SOC and the newly introduced band inversion generates strong spin Berry curvature, resulting in significant intrinsic SOTs. Our results indicate that SOC in materials accompanied by only a minute displacement of light‐element atoms in single crystals can induce strong SOTs, which are beneficial for spin‐orbitronics applications. This finding provides a new guiding principle, which utilizes very local breaking of the crystalline symmetry, for designing materials with substantial SOTs.

## 2 Results and Discussion

### 2.1 Sample Preparation and Characterization

We grew an epitaxial SRO film with a thickness of 26 nm on an STO (001) substrate using a machine learning‐assisted molecular beam epitaxy (MBE) system.^[^ ^34^ ^]^ We made a crossbar device using photolithography and Ar ion milling, followed by sputtering of the Ag electrodes as heat sinks (see Figure 1a and Section 4). For the studied SRO film, the residual resistivity ratio (RRR)^[^ ^35^ ^]^ is defined as the ratio of the resistance at a temperature *T* of 300 K to that at a *T* of 3.7 K and is ≈15.5. The longitudinal resistivity (*ρ_xx_*) shows a kink at *T* ≈ 150 K, which corresponds to the Curie temperature (*T*_C_) (Figure 1b). The SRO film has perpendicular magnetic anisotropy (PMA), as shown by the rectangular hysteresis of the anomalous Hall effect (AHE) in Figure 1c, where the height of the loop is 0.51 Ω at *T* = 90 K. The Hall resistance *R*_H_ is negatively proportional to the perpendicular component of magnetization in the temperature range from *T* = 3.7 to 120 K (see Text S1 and Figure S3, Supporting Information).

### 2.2 Single‐Layer SOT Magnetization Switching in SRO

**Figure 2:**
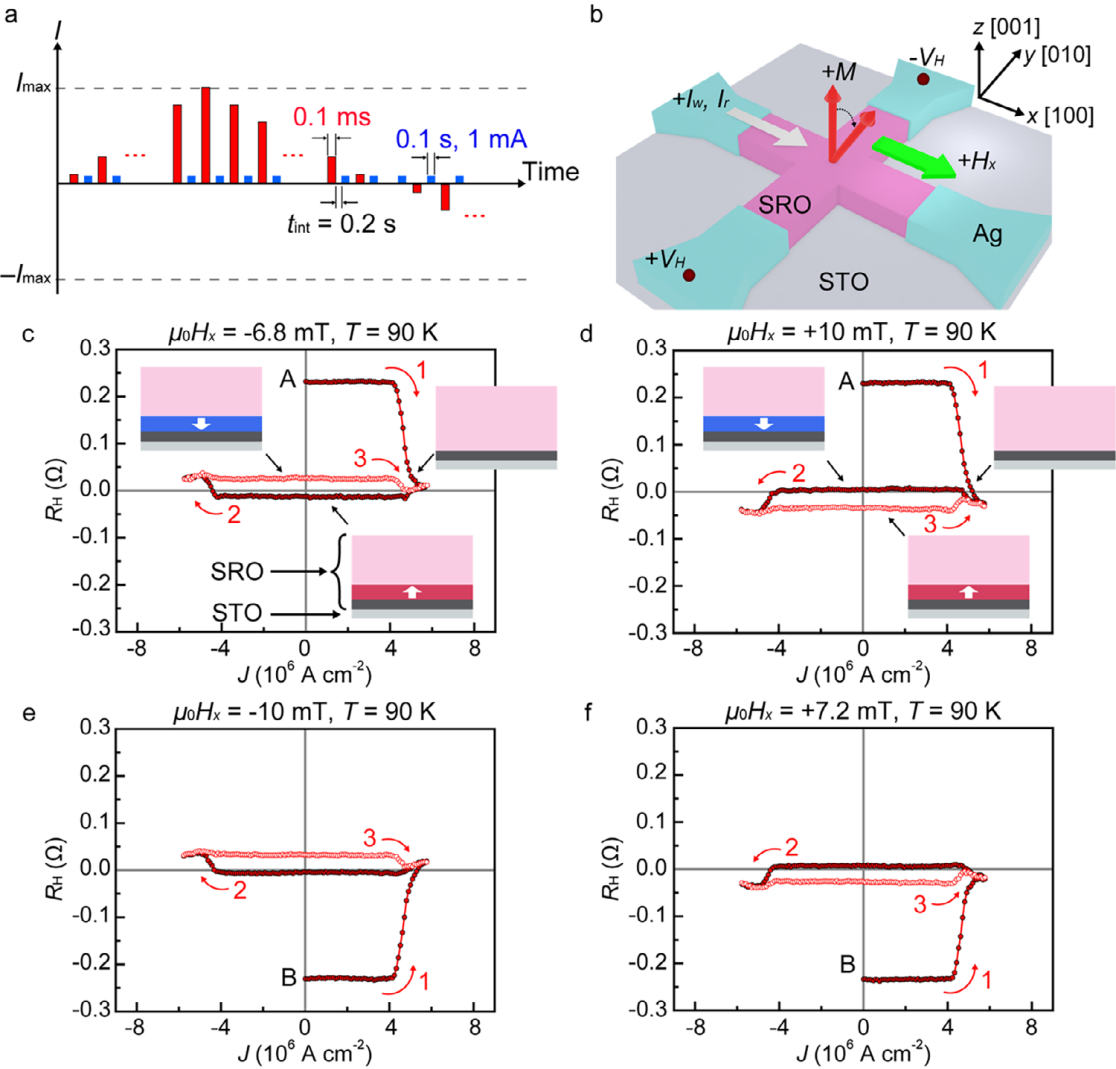
a) Sequence of the SOT magnetization switching measurements. b) Measurement configuration of SOT magnetization switching. The initial magnetization slightly tilts in the *x* direction due to the supporting field *H_x_*. c–f) *R*_H_–*J* loops obtained at *T* = 90 K. Inserted schematic illustrations are the side view of schematic magnetization alignment in the SRO/STO heterostructure corresponding to the indicated points in processes 1, 2, and 3. The gray region represents the STO substrate. The red and blue regions denote magnetic domains with the upward and downward magnetization directions, respectively. These regions correspond to ≈8% of the 26 nm‐thick SRO film near the SRO/STO interface, where the magnetization is likely switched in processes 2 and 3 at 90 K. The pink region is the mixed region of the upward and downward magnetization domains. The dark gray region represents the dead layer of the SRO film.

We performed current‐induced SOT magnetization switching measurements in the following steps. Before the measurements, we applied a large external magnetic field of 1 T along the –*z* (//$\left[00\bar{1}\right]$) or +*z* (//[001]) direction (in pseudo‐cubic notation) to align the spontaneous magnetization in those directions and then decreased the magnetic field to zero. Due to the negative AHE coefficient, the initial state with the magnetization orientation along the –*z* direction corresponds to point A in **Figure** **2**c,d. The initial state of the +*z* magnetization direction corresponds to point B in Figure 2e,f. We applied a weak external magnetic field *H_x_* along the *x* direction to ensure deterministic magnetization switching^[^ ^4^, ^6^, ^9^ ^]^ (Figure 2b). Under continuous application of *H_x_*, a short‐pulsed writing current *I*_w_, whose current density is *J*, with a pulse width of 0.1 ms, was applied in the *x* direction (red squares in Figure 2a). Then, following an interval *t*_int_ of 0.2 s with no current flow, we applied a pulsed reading current *I*_r_ of 1.0 mA with a pulse width of 0.1 s (blue squares in Figure 2a) in the *x* direction. By measuring the *R*_H_ under the application of *I*_r_, we detected the magnetization state of the SRO film.

An important finding in our study is the appearance of *single‐layer* SOT‐induced switching of the magnetization state (Figure 2c–f). As shown in Figure 2c,d, when *J* increases in the positive direction from the initial point A in process 1, *R*_H_ undergoes a sudden jump to an *R*_H_ value of ≈0 Ω at a current density *J* of ≈5 × 10^6^ A cm^−2^. The SRO films grown on STO substrates often exhibit a stripe‐like pattern of ferromagnetic domains with alternating ±*z* magnetization orientations,^[^ ^36^, ^37^ ^]^ reflecting the atomic steps of the TiO_2_‐terminated STO (001) surface. When these multidomain structures dominate in the film, the *R*_H_ approaches ≈0 Ω due to magnetization cancellation. We propose that process 1 likely induces a similar multidomain structure, potentially arising from magnetization instability during current flow.^[^ ^38^ ^]^ Subsequent current reversals (processes 2 and 3) lead to a hysteresis loop in *R*_H_ (Figure 2c,d). Similar behavior is observed starting from the initial state B (Figure 2e,f). Thereafter, the *R*_H_ follows the same hysteresis loop when repeating processes 1, 2, and 3 (see Figure S4, Supporting Information). The slight shift in the center of the hysteresis loops from an *R*_H_ value of 0 depends on *H_x_* and is caused by the small deviation of *H_x_* from the in‐plane direction (≈6°; see Text S2 and Figure S5, Supporting Information). The key feature of our result is the polarity change of the hysteresis loops depending on the sign of *H_x_*; this feature is a hallmark of deterministic SOT magnetization switching.^[^ ^6^, ^9^ ^]^ The loop height is always ≈0.039 Ω, and thus ≈8% (= 0.039 Ω/0.51 Ω) of the total magnetization of the SRO film is stably reversed by the SOTs induced by the in‐plane current. As described later, this partial magnetization switching can be attributed to the octahedral rotations near the SRO/STO interface, leading to magnetization reversal primarily near this interfacial region (insets in Figure 2c,d).

**Figure 3:**
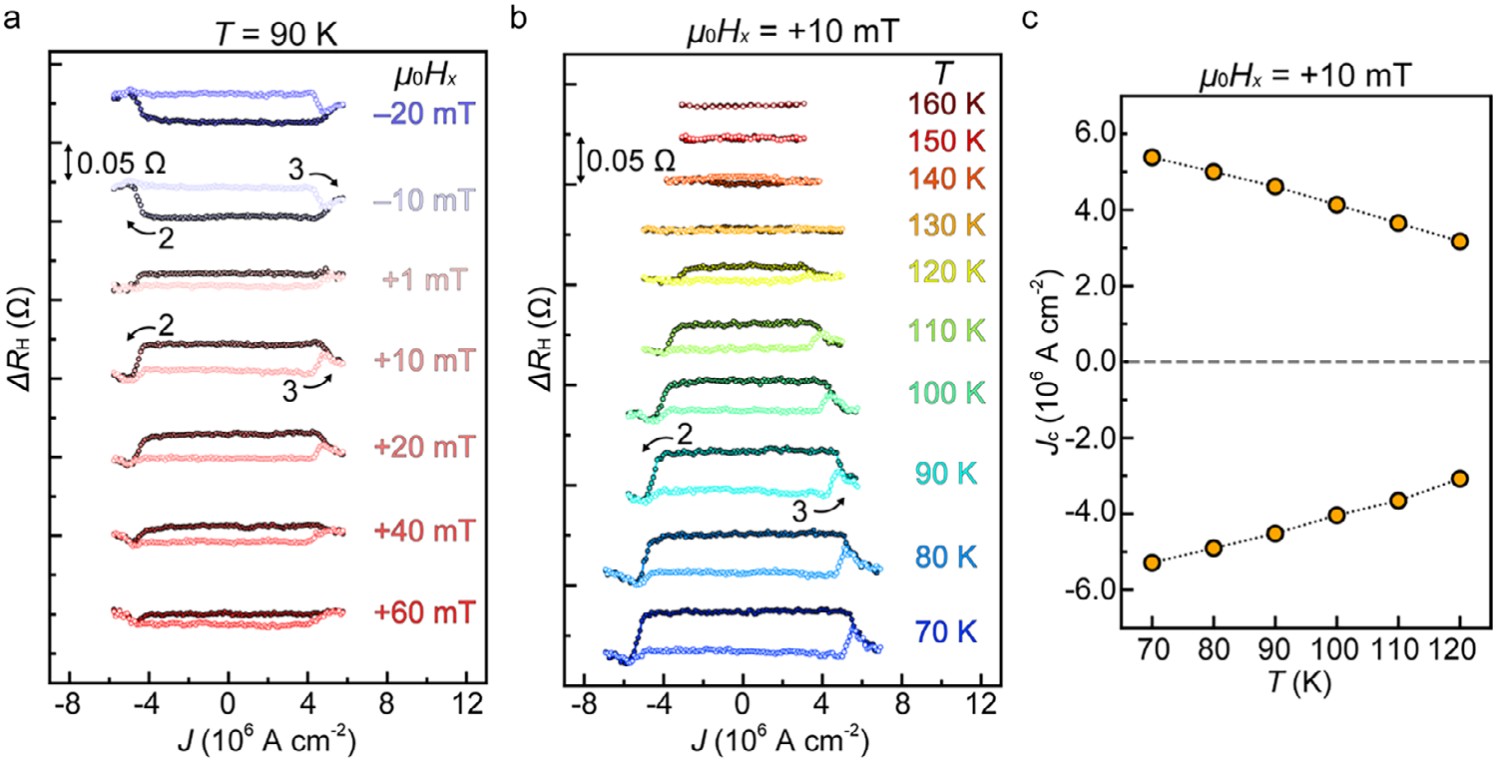
a) Δ*R*_H_–*J* loops under various *H_x_* values at 90 K. b) *ΔR*_H_–*J* loops at various *T* under *µ*_0_ *H_x_* = +10 mT. In (a) and (b), the dark‐colored (pale‐colored) line in each loop corresponds to process 2 (3), as shown in Figure 2c–f. The arrows express the sweep directions. Before each measurement, the magnetization is initialized by applying a strong external magnetic field of 1 T along the –*z* direction, which corresponds to point A in Figure 2c,d. Here, process 1 is not shown, and only processes 2 and 3 are shown. The *ΔR*_H_ obtained by subtracting the average value of the *R*_H_ of each loop from the *R*_H_ is plotted. c) Critical switching current density *J*_c_ as a function of *T* under a *µ*_0_ *H_x_* of +10 mT.

Δ*R*_H_ is defined as the *R*_H_ relative to the center of the hysteresis loops during processes 2 and 3; as shown in **Figure** **3**a, when *H_x_* increases, the hysteresis loop height initially increases but then decreases with increasing *μ*_0_ *H_x_* (≥+40 mT); this phenomenon is caused by the magnetization tilting in the *H_x_* direction and is typical for SOT switching. The switching phase diagram of the relationship between the critical switching current density *J*_c_ and *H_x_* also shows a typical feature of SOT switching, where |*J*_c_| tends to decrease with increasing |*H_x_* | (see Figure S6, Supporting Information). In Figure 3b, we observe the same counterclockwise *R*_H_–*J* loops for *μ*_0_ *H_x_* = +10 mT up to 120 K. The SOT magnetization switching loop disappears at *T* above a *T*_C_ of ≈150 K. As shown in Figure 3c, *J*_c_ decreases with increasing temperature; this result is attributed to the reduced saturation magnetization and weakened magnetic anisotropy (see Figure S2, Supporting Information). Thermal fluctuation may also contribute to the decrease in *J*_c_ with increasing temperature by helping the magnetization to overcome the energy barrier of the switching. The smallest critical switching current density obtained in this study is ≈3.1 × 10^6^ A cm^−2^ at *T* = 120 K.

### 2.3 Broken Inversion Symmetry Due to Minute Displacement of the Oxygen Atoms

**Figure 4:**
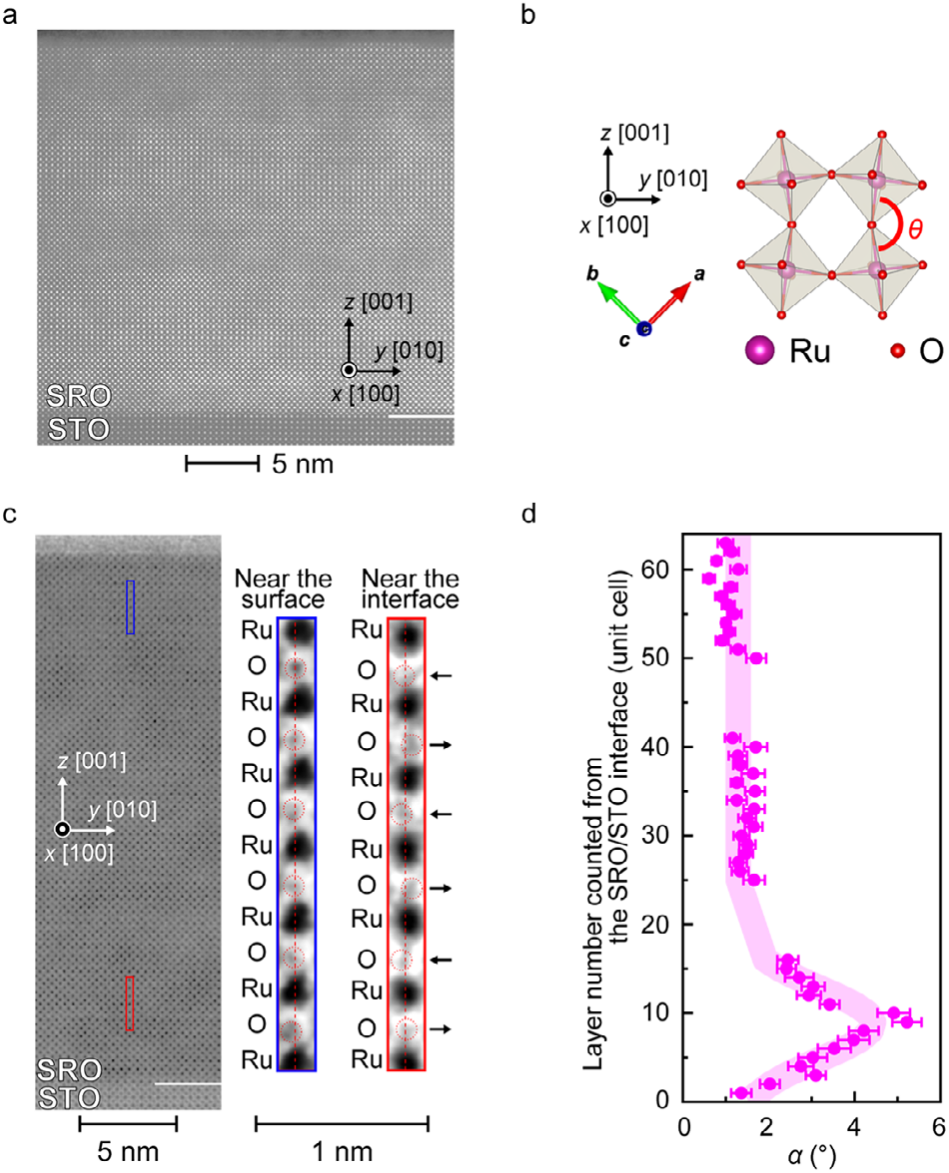
a) High‐angle annular dark‐field (HAADF‐) STEM image of the SRO/STO (001) heterostructure. b) Lattice structure of RuO_6_ octahedrons.^[^ ^47^ ^]^ The coordinates are the same as those in Figure 2b. The arrows colored red, light green, and blue are the crystal axes of SRO on the STO (001) substrate. c) ABF‐STEM image of the SRO/STO (001) heterostructure. The right images are magnified views of the area within the blue and red frames in the main image. Near the SRO/STO interface, the O atoms are displaced alternately along the ±*y* direction. This provides evidence that oxygen octahedral rotation occurs. d) Distribution of the octahedral rotation angle *α* along the direction perpendicular to the film. The 0‐th layer is defined as the oxygen atoms at the SRO/STO interface. The thick light pink line behind is a guide for the eyes.

In general, for single‐layer SOT magnetization switching, we need to break the IS. In our heterostructure, however, no inversion asymmetry appears inside our high‐quality SRO film, as shown in the HAADF‐STEM image in **Figure** **4**a. Hence, to clarify the cause of the observed partial magnetization switching, we need a more precise analysis of the local crystal structure, which is not discernible in Figure 4a. For this purpose, we utilized ABF‐STEM to detect the position of the oxygen atoms (Figure 4c). We find that the oxygen atoms are slightly shifted alternately in the ±*y* direction, especially near the STO interface; these results indicate that RuO_6_ octahedral rotations around the *x*‐axis occur (see arrows in the inset of Figure 4c). The octahedral rotation of oxygen is sensitive to the epitaxial strain,^[^ ^39^, ^40^ ^]^ oxygen vacancies, and interfacial coupling with the octahedra of substrates.^[^ ^41^ ^]^ The rotation magnitudes differ depending on the *z* position within the film. Here, using the bond angle *θ* of Ru─O─Ru (Figure 4b), we define the oxygen octahedral rotation angle *α* around the *x*‐axis as *α* = (180° − *θ*)/2. We find that *α* sharply increases to ≈5° near the SRO/STO interface (Figure 4d). As shown below, the increase in *α* enhances the SHE and SOT, as also demonstrated in ref. [28] using Co/SRO bilayers. We observe a stronger SHE for our single SRO layer, leading to magnetization switching, than that obtained for the bilayers. This result may be attributed to the absence of heterointerfaces, which cause imperfect spin transparency. As discussed later, magnetization switching is considered to occur in the 10–14th unit‐cell layers counted from the SRO/STO interface just above the peak of *α* (=9th layer).

### 2.4 Spin Berry Curvature Generated by the Oxygen Octahedral Rotations

**Figure 5:**
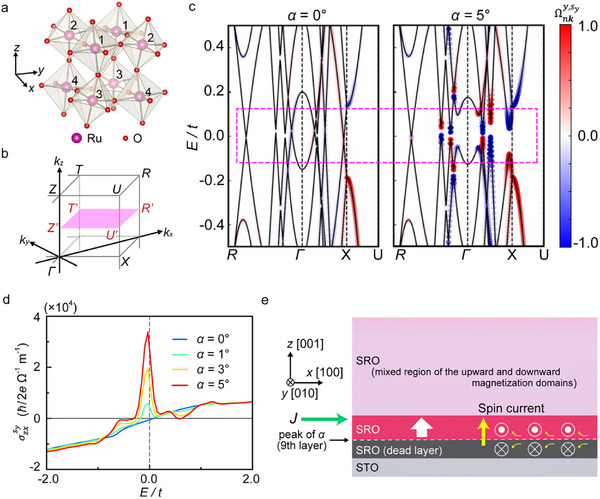
a) Illustration of the sublattices. b) Definition of the symmetric points in the ***k***‐space. Each highly symmetric point corresponds to the Brillouin zone of the orthorhombic unit cell of SRO. (*k_x_*, *k_y_*, *k_z_*) is defined from the crystal axis of the pseudo‐cubic unit cell of SRO (*a’*, *b’*, *c’*). c) Band structure and spin Berry curvature $\Omega_{nk}^{y,s_{y}}$ of SRO when the octahedral rotation angle *α* = 0° and *α* = 5°. The color of each dot displays the value of $\Omega_{nk}^{y,s_{y}}$ rescaled to the range from −1 to +1. The rescaling from its physical value defined by Equation (1), having the dimension of ℏ · m^2^, is obtained by multiplying $\Omega_{nk}^{y,s_{y}}$ by the factor 2/ℏ*a'c'*. d) Intrinsic contribution of spin Hall conductivity (SHC) for various *α*. e) Illustration of the spin Hall effect and induced magnetization switching near the SRO/STO interface. The red region represents the upward magnetization region, where the SOT switching occurs. Here, the spin directions along the *y*‐axis for the positive writing current are illustrated with white marks. The pale pink region is the mixed region of the upward and downward magnetization domains.

To understand the influence of the octahedral rotations, we theoretically calculated the spin Berry curvature and spin Hall conductivity (SHC) in SRO with the tilted RuO_6_ octahedra. The octahedral crystal field splits the Ru 4*d* bands into high‐energy *e_g_* and low‐energy *t*_2_ *_g_*  states. In SRO, electrons exist only in the *t*_2_ *_g_*  band. Hence, we constructed a tight‐binding model,^[^ ^29^, ^42^ ^]^ with six bases of three *t*_2_ *_g_*  orbitals with up and down spins (see Section 4). We considered the band structure of SRO near the SRO/STO interface, where half of the *t*_2_ *_g_*  band is filled because of the charge transfer of one electron from Ru to Ti near the SRO/STO interface.^[^ ^43^ ^]^ Here, Ru^4+^ in SRO has four electrons in the *d* orbital, whereas Ti^4+^ in STO has none; these configurations are energetically favorable for the transfer of an electron from SRO to STO. This model can successfully reproduce the low‐energy band structure, including the Weyl point structure, and the spontaneous magnetization of SRO.^[^ ^29^ ^]^ In addition, neighboring octahedrons located in the same *xz* plane rotate in opposite directions at the same angle. Hence, we consider a unit cell consisting of four sublattices 1–4, as shown in **Figure** **5**a. Since the volume of the unit cell quadruples, the Brillouin zone is folded into a quarter of that for a single octahedron unit cell, by which band crossings appear at highly symmetric points (see Text S3 and Figure S6, Supporting Information). In general, the oxygen octahedrons also rotate around the *z*‐axis in addition to the *x*‐axis; however, the *z*‐axis rotation does not significantly change the calculation results (see Figure S7, Supporting Information). Thus, we consider rotation only around the *x*‐axis hereafter.

To understand the spin Hall conductivity, we calculated the spin Berry curvatures when *α* = 0° and *α* = 5° (Figure 5c,d). We define *E* and *t* as the electron energy and electron hopping amplitude of the *π* bonding ($t_{\pi }^{ij}$) between the nearest neighbor Ru sites, respectively (see Experimental Section). Using the *n*‐th eigenvalue $\varepsilon_{nk}$ and eigenstate $|u_{nk}\rangle$ of the tight‐binding Hamiltonian $H_{k}$ at each wave vector ***k***, we can calculate the spin Berry curvature $\Omega_{nk}^{y,s_{y}}$, defined as follows:

(1) Ωnky,sy=−2∑m(≠n)Imunkjzksyumkumkvkxunkεnk−εmk2

where $v_{k}^{g=x,y,z}$ is the velocity operator expressed as $\partial H_{k}/\partial k_{g}$ and the $j_{zk}^{s_{y}}$ is spin‒current operator defined as $\frac{1}{2}\left\{s_{y},v_{k}^{z}\right\}$. Here, $\left\{s_{y},v_{k}^{z}\right\}$ is an anticommutator, where *s_y_* is the spin operator in the *y* direction and $v_{k}^{z}$ is the velocity operator in the *z*‐direction. As shown in Figure 5c, *E* = 0 is defined as the Fermi level (*E*_F_) position corresponding to the half‐filled state of the *t*_2_ *_g_*  band (see Section 4). At each band crossing obtained when *α* = 0° near the *E*_F_, a small gap opens when *α* = 5° (especially near the X point, we can see significant changes). Simultaneously, around the opening gaps, band inversion appears, leading to a sharp variation in the wave function in ***k***‐space. As a synergetic effect with these band modulations, the inherently existing SOC generates *hot spots* of the spin Berry curvature $\Omega_{nk}^{y,s_{y}}$ (Figure 5c). Accordingly, $\Omega_{nk}^{y,s_{y}}$ is dramatically increased around these gaps.

To understand the spin Berry curvature distribution around the *E*_F_, we examine the Fermi contours in the ***k*** plane of *Z'U’R'T’* (Figure 5b) and the total spin Berry curvature $\Omega_{k}^{y,s_{y}}=\sum_{n}f\left(\varepsilon_{nk}\right)\Omega_{nk}^{y,s_{y}}$, where $f(\varepsilon_{nk})$ is the Fermi distribution defined as $f\left(E\right)={\left[e^{E/T}+1\right]}^{-1}$. Here, we take the limit *T* = 0 K. With increasing *α*, we observe an increase in $\Omega_{k}^{y,s_{y}}$ around the Fermi contours (see Text S3 and Figure S8, Supporting Information). Based on a more detailed analysis of the bands, the spin‐up and spin‐down states are hybridized near the small gaps (see Text S3 and Figure S9, Supporting Information). These gaps cannot be observed in the absence of octahedral rotations, where the band crossings are protected by sublattice symmetry. Once the sublattice symmetry is broken by the octahedral rotations, the hybridization of the opposite spin bands generates *hot spots* of the spin Berry curvature, where the direction of spins sharply varies in ***k***‐space, as the synergetic effect with the SOC.

We derive the intrinsic SHC $\sigma_{zx}^{s_{y}}$, which is defined as the spin‐current density with the *y*‐component spin flowing in the +*z* direction divided by the electric field applied in the *x* direction. By using the Kubo formula, $\sigma_{zx}^{s_{y}}$ is defined as the integral of the spin Berry curvature over the entire Brillouin zone:

(2) $$\sigma_{\mathrm{zx}}^{s_{y}}=e\sum_{n}\int_{\mathrm{BZ}}\frac{d^{3}k}{{\left(2\pi \right)}^{3}}f\left(\varepsilon_{nk}\right)\Omega_{nk}^{y,s_{y}}$$

$\sigma_{zx}^{s_{y}}$ has a sharp peak near *E*_F_ (*E*/*t* = 0 in Figure 5d). With increasing *α*, the magnitude of the peak of $\sigma_{zx}^{s_{y}}$ becomes larger. From our experimental results, the maximum value of $\sigma_{zx}^{s_{y}}$ is estimated to be ≈6.2 × 10^5^ (ℏ/2e) Ω^−1^ m^−1^ at 90 K by using the relations of $\sigma_{zx}^{s_{y}}=\left(\hbar /2e\right)\;\sigma_{xx}\theta_{\mathrm{SH}}$ and $\theta_{\mathrm{SH}}=2eM_{s}t_{\mathrm{FM}}H_{c}/\hbar J_{c}$,^[^ ^44^ ^]^ where *σ*_*xx*_, *θ*_SH_, *M*_s_, *t*_FM_, and *H*_c_ are the longitudinal conductivity, spin Hall angle, saturated magnetization, the thickness of the FM region where magnetization switching occurs, i.e., ≈8% of the 26 nm‐thick SRO film (dark red region in Figure 5e), and coercive field, respectively (see Text S4, Supporting Information). The experimentally estimated value of SHC is larger than the value obtained by the tight‐binding calculation. We discuss possible reasons for this difference in Text S4, Supporting Information. The orbital Hall effect (OHE) and Joule heating are possible contributing factors. Since the OHE in SRO is difficult to quantify accurately, we cannot exclude its contribution to the observed large SHC. Clarifying the role of OHE in SOT‐induced magnetization switching in SRO single layers remains a future issue. Additionally, Joule heating may assist the magnetization switching process by raising the sample temperature (by ≈20 K, see Text S4, Supporting Information), making the experimentally estimated SHC appear larger than the theoretical value.

### 2.5 Mechanism of the Unique Switching Behavior

Figure 5e shows the predicted mechanism of the observed partial SOT magnetization switching. As shown in Figure 2d,f, the magnetization is switched from upward to downward for *J* > 0 when *μ*_0_ *H_x_* > 0, indicating that the damping‐like torque by the –*y*‐polarized spin is generated when *J* > 0.^[^ ^6^ ^]^ Based on the theoretical analysis shown above, a large spin current is generated from around the 9th layer from the SRO/STO interface, where the largest octahedral rotation occurs. Due to the positive sign of $\sigma_{zx}^{s_{y}}$ (see Figure 5d), the spin current diffusing upward has –*y*‐direction spin polarization, exerting a large SOT on the magnetization in the 10–14th layers counted from the SRO/STO interface. This estimation is consistent with the region volume estimated from the SOT magnetization switching experiments shown in Figure 2c–f (≈8% of the 26 nm‐thick SRO film). Similar partial magnetization switching has also been discussed in a ferromagnet with an intentionally controlled composition gradient.^[^ ^23^ ^]^ The region closer to the SRO/STO interface shown in dark gray in Figure 5e is considered a dead layer of ≈3 nm (≈8 MLs) with low conductivity, less magnetization, and magnetization instability; this dead layer does not contribute to deterministic switching, as discussed in Ref. [45], where magnetization switching does not occur. Consequently, we obtain partial SOT‐induced magnetization switching. We further perform the same SOT magnetization switching experiment for a different SRO single‐layer sample with a reduced thickness, finding that the switching ratio is increased, which supports our model (see Text S5 and Figure S11, Supporting Information). Based on this result, even a minute spontaneous displacement of oxygen atoms, as small as ≈0.01 nm, in our film triggers dramatic band modulation and leads to a substantial intrinsic SHE capable of *single*‐layer switching of the magnetization states of ferromagnets.

## 3 Summary

We experimentally demonstrate efficient current‐induced partial SOT magnetization switching in an epitaxial single layer of the oxide Weyl ferromagnet SRO. The inhomogeneous distribution of the spontaneous oxygen octahedral rotations, which are unique to complex oxides, leads to pronounced SOTs in a single SRO ferromagnetic layer. We obtain a small critical switching current density of ≈3.1 × 10^6^ A cm^−2^. The band inversion appears around the small gaps generated at band crossings near the SRO/STO interface due to the broken sublattice symmetry by the octahedral rotations. The inherent SOC in SRO accompanied by this phenomenon results in strong spin Berry curvature, and thus a large intrinsic SHC due to the generation of *hot spots* of spin Berry curvature. Our findings highlight the immense potential for achieving giant SOTs through precise atom positioning in single crystals, unlocking a crucial pathway toward efficient and functional material systems for spin‐orbitronics applications.

## 4 Experimental Section

### Sample Preparation

A 26 nm‐thick SRO film was grown on an STO (001) substrate using a custom‐designed MBE setup equipped with multiple e‐beam evaporators for Sr and Ru.^[^ ^34^ ^]^ For growth, the elemental fluxes were precisely controlled by monitoring the flux rates with an electron‐impact‐emission‐spectroscopy sensor and feeding the results back to the power supplies for the e‐beam evaporators. Oxidation during growth was carried out using a mixture of oxygen (85%) and ozone (15%) gases; these gases were introduced through an alumina nozzle pointed at the substrate. Further information on the MBE setup and preparation of the substrate is described elsewhere. The clear Laue fringes obtained from X‐ray diffraction (Figure S1, Supporting Information) and HAADF‐STEM images shown in Figure 4a indicate the high crystallinity, a large coherent volume of the SRO film, and an abrupt interface between the SRO film and the STO substrate.

### Device Preparation and Electrical Measurements

The SRO/STO sample was patterned into a crossbar device with a channel width and length of 10 and 40 µm, respectively, via photolithography and argon ion milling. Afterward, Ag was sputtered to produce four electrode terminals, which also functioned as heat sinks. From the kink observed in the temperature dependence of the resistivity *ρ_xx_*, the Curie temperature of the device was estimated to be ≈150 K. For the SOT magnetization switching measurements, a Keithley 6221A was used as a pulsed current source.

### Theoretical Calculations Based on a Tight‐Binding Model

A tight‐binding model was constructed for SRO, following the schemes in Refs. [29, 42]. The band structure of SRO was considered near the SRO/STO interface, where half of the *t*_2_ *_g_*  band is filled because of the charge transfer of one electron from Ru to Ti near the SRO/STO interface.^[^ ^43^ ^]^ Here, Ru^4+^ in SRO has four electrons in the *d* orbital, whereas Ti^4+^ in STO has none; these configurations are energetically favorable for the transfer of an electron from SRO to STO. This tight‐binding model effectively reproduces the low‐energy band structure, including the Weyl point structure under spontaneous magnetization. The band structure of SRO was calculated considering the nearest neighbor (NN) and next‐nearest neighbor (NNN) direct Ru‐Ru hopping, where the states with effective total angular momentum *J*_eff_ values of 1/2 and 3/2 are mixed. The tight‐binding Hamiltonian $H_{k}$ consists of the following four terms:

(3) $$H_{k}=H_{k}^{\mathrm{NN}}+H_{k}^{\mathrm{NNN}}+H_{k}^{\mathrm{SO}}+H_{k}^{\mathrm{exc}}$$

Here, $H_{k}^{\mathrm{NN}}$ and $H_{k}^{\mathrm{NNN}}$ represent the hopping between the NN and NNN sites, respectively. $H_{k}^{\mathrm{SO}}$ denotes the spin‒orbit coupling at each site. $H_{k}^{\mathrm{exc}}$ represents the effect of spin splitting by spontaneous magnetization.

*α* and *γ* are defined as the angles of rotation of a RuO_6_ octahedron around the *x*‐axis and around the *z*‐axis, respectively. Because the neighboring octahedrons located in the same *xz* plane rotate in opposite directions at the same angle, a unit cell is composed of four sublattices (1, 2, 3, and 4) of Ru sites, as shown in Figure 5a. Note that, for *α* = 0°, the pseudo‐cubic unit cell of SRO was considered at first, whose lattice parameters are *a*′  =  *b*′  =  *c*′  = 3.93 nm;^[^ ^46^ ^]^ these values are different from those of the orthorhombic unit cell (in the case of *α* > 0°).

As the basis for the Hamiltonian, three *t*_2_ *_g_*  orbitals (*d_yz_*,*d_zx_*,*d_xy_*) was utilized. Due to octahedral rotations, the crystal field is rotated in each sublattice, and thus, the directions of the *t*_2_ *_g_*  orbitals are different among the sublattices. Thus, to introduce octahedral rotations into the model, the local coordinate (*x*′, *y*′, *z*′) was taken for each sublattice (see details in Text S3, Supporting Information).

The spin Berry curvature $\Omega_{nk}^{y,s_{y}}$ arises around the band inversion points, where the band spacing $\left(\varepsilon_{nk}-\varepsilon_{mk}\right)$ becomes small and the Bloch wave function sharply varies in the ***k***‐space. In the same manner, the dimensionless total spin Berry curvature $\Omega_{k}^{y,s_{y}}$ is mapped to Figure S8 (Supporting Information) and shown in the range from −1 to +1 after scaling by applying the factor 2/ℏ*a*′*c*′ to the original value.

### Details of the Scanning Transmission Electron Microscopy (STEM) Measurements

The sample used for the STEM measurement (for obtaining Figure 4) was prepared using an NX2000 (Hitachi High‐Tech. Co.) triple‐beam focused‐ion‐beam (FIB) scanning electron microscope (SEM) system. The sample was milled by a Ga‐ion beam, followed by the irradiation of an Ar‐ion beam to remove a damaged surface layer. Prior to FIB milling, a carbon (≈200 nm) was deposited on the sample surface as a protective layer. Both HAADF‐ and ABF‐STEM images were acquired using JEM‐ARM200F (JEOL Ltd.) with an acceleration voltage of 200 kV at room temperature. The collection‐angle ranges for HAADF and ABF imaging were 68–280 and 10–34 mrad, respectively. The images were processed using Gaussian Blur (for reducing noise) and Unsharp Masking (for enhancing edges and increasing contrast).

A detailed STEM analysis was also performed to confirm that the positions of the Sr and Ru atoms hardly fluctuate (see Figure S12, Supporting Information), as follows. The sample used for this STEM analysis was prepared using an NB5000 (Hitachi High‐Tech. Co.) dual‐beam FIB‐SEM system. The sample was milled by a Ga‐ion beam with an energy of 40 keV, followed by milling with lower beam energies of 5, 2, and 1 keV to reduce beam irradiation damage. The HAADF‐STEM observation was performed using JEM‐ARM200F (JEOL Ltd.) with an acceleration voltage of 200 kV at room temperature. The convergence semi‐angle for the electron probe was ≈26 mrad, and the collection‐angle range for HAADF imaging was 90–270 mrad. Ten frames of the region of interest were acquired and averaged using non‐rigid registration with SmartAlign^[^ ^48^ ^]^ (HREM Research Inc.) to improve the signal‐to‐noise ratio. Each frame contains 1024 × 1024 pixels, with each pixel corresponding to an area of ≈10 × 10 pm^2^. The pixel dwell time and flyback time were 2 and 500 µs, respectively. Atomic positions in the multi‐frame averaged STEM images were determined using 2D Gaussian fitting. To ensure accurate lattice parameter measurements, distortion of the STEM images caused by sample drift and the scanning system of the microscope were corrected using a two‐step affine transformation method.^[^ ^49^ ^]^

Those STEM measurements were carried out at room temperature. Meanwhile, the SOT magnetization switching measurements were carried out at temperatures below 160 K. Possible phenomena that may significantly affect the oxygen octahedral rotation in SRO when changing temperature are the structural phase transition (SPT) of SRO itself and temperature‐dependent strain from the SrTiO_3_ (STO) substrate via the SPT of STO. However, the influences of the SPT of SRO and STO are insignificant, as described in Text S6 (Supporting Information).

### Statistical Analysis

All statistical data (Figure 4d; Figure S12c) are presented in the form of (mean value) ± (standard error). The lattice parameters were estimated by averaging *n* STEM lattice images, where *n* = 19 (for *α* of 1−16th layers in Figure 4d), *n* = 19 (for *α* of 25−41th layers in Figure 4d), *n* = 20 (for *α* of 50−63th layers in Figure 4d), *n* = 77 (for *a* and *c* of the −4th to the 18th layers in Figure S12b, which was estimated based on Figure S12a, Supporting Information), and *n* = 71 (for *a* and *c* of 37−58th layers in Figure S12b, which was estimated based on Figure S12a, Supporting Information). No pre‐processing of data/statistical tests was conducted in this study.

## Conflict of Interest

The authors declare no conflict of interest.

## Author Contributions

H.H. and Y.K.W. performed sample preparation. Y.K.W., Y.T., H.Y., and Y.K. performed sample growth. H.H. performed measurements. H.H. and S.K‐T. performed data analysis. Y.A. performed calculations. Y.A., J.I., and M.Y. performed theoretical modeling. Y.S. performed STEM analyses. H.H., Y.A., Y.K.W., M.T., and S.O. performed writing and project planning.

## Supporting information



Supporting Information

## Data Availability

The data that support the findings of this study are available from the corresponding author upon reasonable request.
