# Supplementary material for: Single‐Layer Spin‐Orbit‐Torque Magnetization Switching Due to Spin Berry Curvature Generated by Minute Spontaneous Atomic Displacement in a Weyl Oxide
Source: Adv Mater. 2025 Apr 24;37(26):2416091. doi: 10.1002/adma.202416091 (PMC12232241; doi:10.1002/adma.202416091)
Supplement: Supplementary file 1 — Supporting Information [file ADMA-37-2416091-s001.pdf]

ADVANCED MATERIALS

Supporting Information

for *Adv. Mater.*, DOI 10.1002/adma.202416091

Single-Layer Spin-Orbit-Torque Magnetization Switching Due to Spin Berry Curvature Generated by Minute Spontaneous Atomic Displacement in a Weyl Oxide

Hiroto Horiuchi, Yasufumi Araki*, Yuki K. Wakabayashi*, Jun'ichi Ieda, Michihiko Yamanouchi, Yukio Sato, Shingo Kaneta-Takada, Yoshitaka Taniyasu, Hideki Yamamoto, Yoshiharu Krockenberger, Masaaki Tanaka* and Shinobu Ohya**

Supporting Information

Single-Layer Spin-Orbit-Torque Magnetization Switching due to Spin Berry Curvature Generated by Minute Spontaneous Atomic Displacement in a Weyl Oxide

Hiroto Horiuchi, Yasufumi Araki, Yuki K. Wakabayashi, Jun'ichi Ieda,
Michihiko Yamanouchi, Yukio Sato, Shingo Kaneta-Takada, Yoshitaka Taniyasu, Hideki Yamamoto, Yoshiharu Krockenberger, Masaaki Tanaka, and Shinobu Ohya

Supporting Text 1: Sample characterizations

The out-of-plane X-ray diffraction (XRD) pattern for the $SrRuO_3$ (SRO)/$SrTiO_3$ (STO) heterostructure shows peaks with Laue (Kiessig) fringes (see Figure S1), confirming that the sample is single phase with an abrupt interface.

The temperature dependence of magnetic moment obtained by a superconducting quantum interface device (SQUID) shows that the Curie temperature (T_C) of the SRO film is around 150 K (see Figure S2), which agrees with the temperature dependence of the resistivity shown in Figure 1b.

The SRO film has perpendicular magnetic anisotropy (PMA), as shown by the rectangular hysteresis loops of the anomalous Hall effect (AHE) (see Figure S3). The Hall resistance R_H is *negatively* proportional to the perpendicular component of magnetization in the temperature range from 3.7 K to 120 K.

Supporting Text 2: SOT-magnetization switching measurements

Before each SOT-magnetization switching measurement, we applied a large external magnetic field $\mu_0 H$ of 1 T along the +z or –z direction to align the magnetization in those directions as an initialization process. As shown in Figure S4a,c,e,g, each measurement starts from the saturated R_H values with the magnetization directions along the –z (point A) and +z (point B) directions.

After one measurement sequence (processes 1, 2, and 3) was completed, we repeated the same processes 1, 2, and 3 under the same in-plane magnetic field H_x without initialization. Here, each measurement started with the R_H value obtained at the end of the previous sequence. In those repeated sequences, we observed the same hysteresis loop as that obtained in the first sequence (Figure S4b,d,f,h). This result indicates that the magnetization is stably switched and that the switching process is non-volatile.

As shown in Figure 2c–f, the center of the R_H – current density (J) loops deviate from R_H = 0 Ω depending on the sign and magnitude of H_x. This offset is due to a slight misalignment of the sample, whose surface is slightly displaced in the xz plane by angle φ from the direction of H_x, as shown in Figure S5a. In fact, with increasing φ in the positive direction from 0°, the offset shifts in the plus direction of R_H for $H_x < 0$ (Figure S5b) and in the minus direction for $H_x > 0$ (Figure S5c). This phenomenon originates from the multidomain region shown as pale pink in Figure 2c,d.

To understand this behavior, we consider a multidomain structure where +z- and –z-oriented magnetization domains are located alternatively, as reported for SRO[S1]. As shown in the lower inset of Figure S5c, when $H_x > 0$ and $\varphi > 0$, the magnetization oriented along the –z direction is more largely tilted towards the x direction than that oriented along the +z direction because the +z direction is closer to the H_x direction than the –z direction. Here, with increasing φ, the magnetization direction changes from the light-colored white arrow to the dark-colored white arrow in the downward magnetization domain. Under this configuration, the magnetization oriented along the +z direction is not significantly affected by the increase in φ (see the upward magnetization domain of the bottom inset in Figure S5c). Thus, the total magnetization of the multidomain region becomes positive. As a result, due to the negative AHE coefficient, the hysteresis loop has a negative offset when $H_x > 0$ and $\varphi > 0$ (Figure S5c). From this measurement, φ is estimated to be 6°. Here, as clearly seen in Figure S5b,c, the shape of the hysteresis loops does not change even though the offset changes. Hence, this misalignment of the sample does not affect the switching process itself.

Figure S6 shows the switching phase diagram of the relationship between J_c and $\mu_0 H_x$. $|J_c|$ tends to decrease with increasing $|H_x|$, which is a typical feature of SOT-induce magnetization switching[S2]. The J_c values are somewhat different from the data shown in Figure 3 in the main manuscript since the device we measured here is different from the one we have studied in the main manuscript, but the sample structure is identical.

Supporting Text 3: Theoretical calculation of the spin Berry curvature and spin Hall conductivity in SRO

We theoretically calculated the spin Berry curvature and the spin Hall conductivity (SHC) based on the scheme in refs. [S3,S4]. We considered the band structure of SRO near the SRO/STO interface, where half of the t_{2g} band is filled due to the charge transfer of one electron from Ru to Ti near the SRO/STO interface[S5].

As the basis for the Hamiltonian shown in the Experimental Section, we utilized the three t_{2g} orbitals $\left(d_{yz}, d_{zx}, d_{xy}\right)$. Due to octahedral rotations, the crystal field direction and, thus, the directions of the t_{2g} orbitals are different among the sublattices. Thus, we considered the local coordinate (x', y', z') for each sublattice. It is related to the original coordinate (x, y, z), which is equivalent to the crystal axes of the pseudocubic SRO cell, (see Figure 5a) as

$$\begin{pmatrix} x' \\ y' \\ z' \end{pmatrix} = \begin{pmatrix} \cos\gamma_i & -\sin\gamma_i & 0 \\ \cos\alpha_i \sin\gamma_i & \cos\alpha_i \cos\gamma_i & \sin\alpha_i \\ -\sin\alpha_i \sin\gamma_i & -\sin\alpha_i \cos\gamma_i & \cos\alpha_i \end{pmatrix} \begin{pmatrix} x \\ y \\ z \end{pmatrix}, \tag{S1}$$

where α_i and γ_i are the rotation angles around the x and the z axes, respectively, in sublattice i (= 1,…,4, see Figure 5a). α_i and γ_i satisfy the following conditions;

$$\alpha_1 = -\alpha_2 = -\alpha_3 = \alpha_4 \equiv \alpha, \qquad \gamma_1 = -\gamma_2 = \gamma_3 = -\gamma_4 \equiv \gamma. \tag{S2}$$

In this coordinate, the t$_{2g}$ orbitals $\left(d_{y'z'}, d_{z'x'}, d_{x'y'}\right)$ are given as the linear combinations of $d_{yz}, d_{zx}, d_{xy}, d_{x^2-y^2},$ and $d_{3r^2-z^2}$ defined in the original coordinate as

$$\begin{pmatrix} d_{y'z'} \\ d_{z'x'} \\ d_{x'y'} \end{pmatrix}$$

$$= \begin{pmatrix} \cos 2\alpha_i \cos\gamma_i & \cos 2\alpha_i \sin\gamma_i & -\frac{1}{2}\sin 2\alpha_i \sin 2\gamma_i & \frac{1}{2}\sin 2\alpha_i \cos 2\gamma_i & \frac{\sqrt{3}}{2}\sin 2\alpha_i \\ -\cos\alpha_i \sin\gamma_i & \cos\alpha_i \cos\gamma_i & -\sin\alpha_i \cos 2\gamma_i & -\sin\alpha_i \sin 2\gamma_i & 0 \\ -\sin\alpha_i \sin\gamma_i & \sin\alpha_i \cos\gamma_i & \cos\alpha_i \cos 2\gamma_i & \cos\alpha_i \sin 2\gamma_i & 0 \end{pmatrix} \begin{pmatrix} d_{yz} \\ d_{zx} \\ d_{xy} \\ d_{x^2-y^2} \\ d_{3r^2-z^2} \end{pmatrix} \tag{S3}$$

We denote this 3×5 matrix as C^i_{lm} (with l = $y'z'$, $z'x'$, $x'y'$ and m = yz, zx, xy, x^2-y^2, $3r^2-z^2$), where the superscript i denotes the sublattice site index. We used this coordinate transformation to construct the tight-binding model with octahedral rotation.

The hopping terms are constructed using Slater-Koster's method. When we consider the hopping between orbital d^i_l on site i and orbital d^j_m on site j (with l, m = yz, zx, xy, x^2-y^2, $3r^2-z^2$), the hopping amplitude t^{ij}_{lm} is given by the linear combination of those of the σ, π, and δ-

bondings, t_{σ}^{ij}, t_{π}^{ij}, and t_{δ}^{ij}, respectively. The structure of t_{lm}^{ij} depends on the orientation of site *i* from site *j*. The hopping amplitude between the t_{2g} orbitals in the local coordinate ($\tilde{t}_{lm}^{ij}$) becomes

$$\tilde{t}_{lm}^{ij} = \sum_{o,p} C_{lo}^{i}\, t_{op}^{ij}\, {}^{t}C_{pm}^{j} \;, \tag{S4}$$

where, the letter *t* on the left shoulder of *C* means the transposed matrix of *C*, and *o* and *p* take *yz*, *zx*, *xy*, $x^2 - y^2$, and $3r^2 - z^2$. We used $\tilde{t}_{lm}^{ij}$ for the NN and NNN hopping terms.

Here, neighboring octahedrons located in the same *yz* plane rotate in opposite directions with the same angle. Hence, we considered a unit cell consisting of four sublattices 1–4 shown in Figure 5a. Since the volume of the unit cell quadruples, the Brillouin zone is folded into a quarter of that for a single octahedron unit cell, by which band crossings appear at and around high-symmetric points, for example, at *T*, *U*, *X*, *Z* and around *Γ* (see red and blue dashed lines in Figure S7).

In general, octahedrons also rotate around the *z* axis in addition to the *x* axis. As seen in Figure S8, increasing only the *z*-axis rotation, whose angle is defined as *γ*, does not significantly enhance the SHC $\sigma_{zx}^{s_y}$ in comparison with the case when increasing only the *x*-axis rotation angle *α*.

The octahedral rotations enhance the total spin Berry curvature, intensifying the spin Hall effect. Figure S9 shows the Fermi contours in the ***k*** plane of *Z'U'R'T'* and the sum of spin Berry curvature $\Omega_{n\boldsymbol{k}}^{y,s_y}$ (color scale) defined as $\Omega_{\boldsymbol{k}}^{y,s_y} = \sum_n f(\epsilon_{n\boldsymbol{k}})\Omega_{n\boldsymbol{k}}^{y,s_y}$, where $f(\epsilon_{n\boldsymbol{k}})$ is the Fermi distribution function. Temperature *T* is set at 0 K. Fermi contours named A that can be seen when $\alpha = 0°$ (bottom left area surrounded by the green broken circle in Figure S9b) are not visible when $\alpha = 5°$ (Figure S9c), indicating that a small gap opens for this band. Fermi contours named B that are very close to each other when $\alpha = 0°$ (upper right area surrounded by the green broken circle in Figure S9b) merge into one when $\alpha = 5°$ (Figure S9c), indicating the splitting bands that cross slightly below the E_F are lifted up due to gap opening by octahedral rotations. By comparing Figure S9b ($\alpha = 0°$) and Figure S9c ($\alpha = 5°$), one can see that $\Omega_{\boldsymbol{k}}^{y,s_y}$ is strongly enhanced around the Fermi contours by octahedral rotations.

Changes above in the electronic structure and the enhancement of $\Omega_{\boldsymbol{k}}^{y,s_y}$ originate from the band repulsion and hybridization because of the broken sub-lattice symmetry by octahedral rotations. Here, we discuss the mechanism of how the octahedral rotation influences the emergence of spin Berry curvature by considering the symmetry of the system. We focus on

the band crossing and repulsion structure around $E = 0$.

In the absence of octahedral rotations, we find many Fermi contours that are almost doubly degenerate around $E = 0$. Such a structure around $E = 0$ can be understood from the "approximate" chiral symmetry and the sublattice symmetry. The dominant parts $\mathcal{H}_{\boldsymbol{k}}^{\mathrm{NN}}$ and $\mathcal{H}_{\boldsymbol{k}}^{\mathrm{exc}}$ in the tight-binding Hamiltonian are antisymmetric under the hypothetically defined unitary transformation $\Gamma = Q s_y$,

$$\Gamma \mathcal{H}_{\boldsymbol{k}}^{\mathrm{NN}} \Gamma^{-1} = -\mathcal{H}_{\boldsymbol{k}}^{\mathrm{NN}}, \qquad \Gamma \mathcal{H}_{\boldsymbol{k}}^{\mathrm{exc}} \Gamma^{-1} = -\mathcal{H}_{\boldsymbol{k}}^{\mathrm{exc}}, \tag{S5}$$

where Q multiplies the phase factor +1 or –1 on each sublattice,

$$Q: (d_1, d_2, d_3, d_4) \to (d_1, -d_2, -d_3, d_4). \tag{S6}$$

Equation (S5) means that Γ serves as the chiral symmetry for $\mathcal{H}_{\boldsymbol{k}}^{\mathrm{NN}}$ and $\mathcal{H}_{\boldsymbol{k}}^{\mathrm{exc}}$. If we consider only $\mathcal{H}_{\boldsymbol{k}}^{\mathrm{NN}}$ and $\mathcal{H}_{\boldsymbol{k}}^{\mathrm{exc}}$, we can rigorously conclude from the chiral symmetry that the states at $E = 0$ are doubly degenerate. Once we introduce the terms $\mathcal{H}_{\boldsymbol{k}}^{\mathrm{NNN}}$ and $\mathcal{H}_{\boldsymbol{k}}^{\mathrm{SO}}$, they slightly violate the chiral symmetry. Nevertheless, since the system still satisfies the sublattice symmetries, which are defined by the half translation of the unit cell,

$$T_y^{1/2}: (d_1, d_2, d_3, d_4) \to (d_2, d_1, d_4, d_3) \tag{S7}$$

$$T_z^{1/2}: (d_1, d_2, d_3, d_4) \to (d_3, d_4, d_1, d_2). \tag{S8}$$

Therefore, the double degeneracies mentioned above are protected by the sublattice symmetries, while their energies are slightly lifted from $E = 0$. We plot the eigenvalues of $T_y^{1/2}$ for each band around $E = 0$, as shown in Figure S10b. We find several bands with $T_y^{1/2} = +1$ and $T_y^{1/2} = -1$ crossing around $E = 0$, which are not gapped out due to the symmetry $T_y^{1/2}$. These crossing bands are almost spin polarized, with $s_z = \uparrow$ and $s_z = \downarrow$, as shown in Figure S10c.

Now we introduce the effect of the octahedral rotations. Since the sublattice symmetry $T_y^{1/2}$ (or $T_z^{1/2}$) protecting the band crossings is broken, the bands with $T_y^{1/2} = +$ and $T_y^{1/2} = -$ are now hybridized and gapped out, as we show in Figure S10e. As a consequence, the bands with $s_z = \uparrow$ and $s_z = \downarrow$ are hybridized, and thus the direction of spins on each band is drastically altered around the hybridization points in momentum space, as shown in Figure S10f. In other words, we can regard that the effect of spin-orbit coupling is magnified drastically around the hybridization points, which forms *hot spots* of spin Berry curvature.

Supporting Text 4: Estimation of the SHC

The ratio of $\sigma_{zx}^{s_y}$ to the longitudinal conductivity σ_{xx} is defined as the spin Hall angle θ_{SH}, which is estimated as

$$\theta_{\mathrm{SH}} = \frac{2e}{\hbar} \cdot M_{\mathrm{s}} t_{\mathrm{FM}} \cdot \frac{H_{\mathrm{c}}}{J_{\mathrm{c}}}, \tag{S9}$$

where e, $\hbar$, M_s, t_{FM}, H_c, and J_c are the elementary charge, reduced plank constant, saturated magnetization, thickness of the switched area in the SRO film (see Figure 5e), coercive field, and the critical switching current, respectively[S6,S7]. Equation (S9) refers to the efficiency of SOT-magnetization switching via local domain wall depinning. By substituting $M_s = 1.42\times10^5$ A m^{-1}, $t_{FM} = 2.08$ nm (= ~ 8 % of the 26 nm-thick SRO film), $H_c = 1800$ Oe, and $J_c = 4.5\times10^6$ A cm^{-2}, all of which are obtained experimentally at $T = 90$ K, to equation (S9), we obtain θ_{SH} ~ 0.57. Thus, from the relation

$$\theta_{\mathrm{SH}} = \left(\frac{2e}{\hbar}\right) \sigma_{zx}^{s_y} \cdot \sigma_{xx}, \tag{S10}$$

we can roughly estimate $\sigma_{zx}^{s_y}$ ~ 6.2×10^5 $(\hbar/2e)$ Ω^{-1} m^{-1} at 90 K. Here, we neglected the effect of the spin diffusion at the interface between ferromagnet and non-magnet that is considered in bilayer systems and is determined by spin transparency[S8]. The estimated value of $\sigma_{zx}^{s_y}$ is about 2.7 times larger than that for a Co/SRO bilayer system[S8].

Additionally, we further discuss other possible constituents of the experimentally determined large SHC, such as the orbital-Hall effect (OHE), Rashba-Edelstein effect (REE), bulk-originated self-induced torque, and Joule heating effect.

At present, precisely estimating the OHE is very challenging. Thus, we are not able to exclude its contribution from the enhanced SHC obtained from the experiments. Clarifying the magnitude of the contribution of the OHE to SOT-induced magnetization switching is a future issue.

The REE and the self-induced torque originating from the bulk SRO region are not so significant in our film. Q. Xie *et al.*[S9] demonstrated SOT-magnetization reversal in a h-BN/SRO/STO sample, in which an extremely thin SRO layer with a thickness of 6 nm was used. Also, they performed the same experiment on the SRO "single layer" film with the same thickness (6 nm) grown on STO; however, *no* switching was observed in this reference experiment, which indicates that the self-induced torque and the REE originating from the SRO/STO interface are *not* significant for the magnetization reversal. Furthermore, such thin SRO films have very small OOR and show neither gradation nor any abrupt change of the OOR

angle[S8,S10]. Thus, in our system, a self-induced torque originating from a spin current generated from the bulk region, where the magnitude of OOR is small and does not have a gradation/abrupt peak, does not play an important role. Also, in our film, the positions of Sr and Ru atoms hardly fluctuate (see Figure S12). Therefore, as we proposed, the most plausible scenario is that the presence of the sharp peak of the angle of OOR causes spin-current generation and magnetization switching.

To examine the contribution of the Joule heating effect, we have estimated the *actual* sample temperature (T_{sw}) when switching occurred. To estimate T_{sw}, we measured the resistivity ρ_{xx} under the application of a writing current pulse with density J (data is not shown), and compared it with the temperature dependence of ρ_{xx} (Figure 1b). As a result, we found that T_{sw} was ~ 110 K when the *set* value of temperature T_{set} was 90 K, meaning that the sample temperature was increased by 20 K due to the writing current pulse. We note that the T_{sw} values obtained at all measured temperatures were always below T_C (= 150 K). Our results imply that the Joule heating assists the SOT magnetization switching (reduces the switching current density J_c) to some extent, which may lead to the experimental SHC that is apparently larger than that obtained by the calculation using the tight-binding model.

Supporting Text 5: SOT magnetization switching in the SRO single layer with a reduced thickness

We performed a SOT magnetization switching experiment for a different SRO single layer with a reduced thickness of 10 nm to validate our model proposed in Figure 5e. Figure S11 shows the AHE and the Hall resistance R_H of this sample as a function of the current density J. The obtained switching ratio, defined as the size of the switching loop (processes 2 and 3 in Figure S11a) relative to that of the AHE loop (Figure S11b), was ~11.7% (= 0.146Ω/1.25Ω). This value is larger than that of the 26 nm-thick film (~8% as shown in Figure 2c–f in the main manuscript). Therefore, as we expected, the portion of the switching area tends to increase with decreasing the film thickness. Although this increase in the switching ratio is not inversely proportional to film thickness, changes in other factors, such as the film quality, the population of pinning sites, and the portion of the region where OOR is large, may affect the switching ratio.

Supporting Text 6: Discussion on the change in the oxygen octahedral rotation depending on the measurement temperature

We carried out the STEM measurements at room temperature and the SOT magnetization switching measurements at temperatures below 160 K. Possible phenomena that may significantly affect the oxygen octahedral rotation (OOR) in SRO when changing temperature are the structural phase transition (SPT) of SRO itself and temperature-dependent strain from the $SrTiO_3$ (STO) substrate via the SPT of STO. SRO films exhibit the SPT from the orthorhombic to the tetragonal phase at ~200 ℃[S11]. Although the SPT temperature depends on the film thickness[S12], it is much higher than room temperature in the range of the film thicknesses including both 10 and 26 nm used in this study. STO exhibits the SPT from cubic to tetragonal phase at 105 K with decreasing temperature[S13]. When further decreasing temperature, the lattice expands (shrinks) along the *c*-axis (*a*-axis)[S14]; however, the lattice-constant change is at most 0.3 pm, which is much smaller than the displacement of the O atoms of ~ 0.01 nm in our system. Indeed, we do not see any abrupt changes due to the SPT of STO in the temperature-dependent behavior of the transport and magnetic properties (Figure 1b and Figure 3c in the main manuscript). Therefore, the influence of the temperature change on the OOR is insignificant.

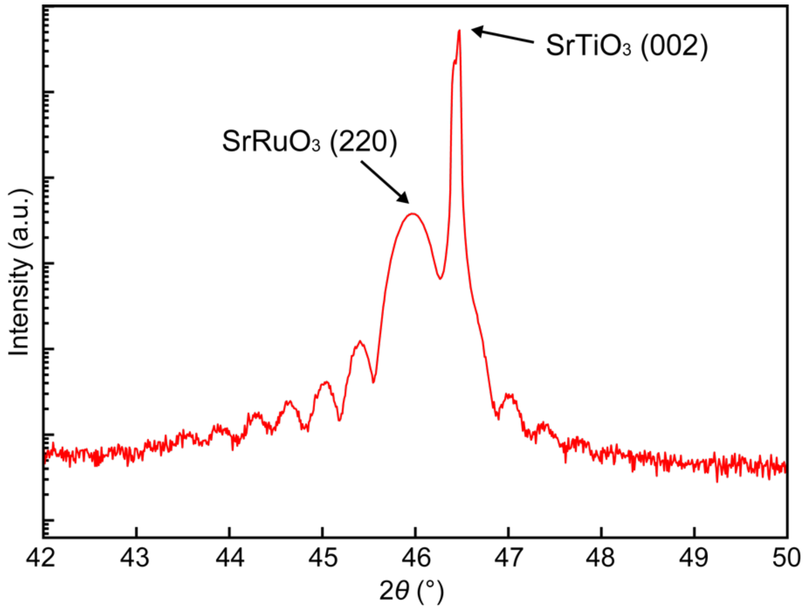

Figure S1. X-ray diffraction (XRD) patterns of SRO on the STO substrate. XRD 2θ–ω scan of SRO (26 nm)/STO is shown.

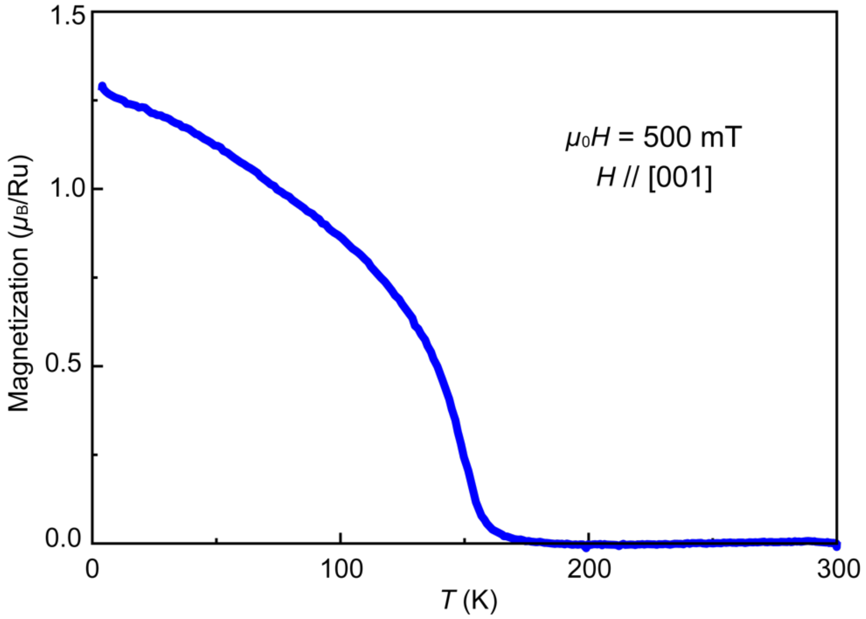

Figure S2. Temperature (T) dependence of the magnetization. The external magnetic field of 500 mT is applied along the z ([001] of the STO substrate) direction.

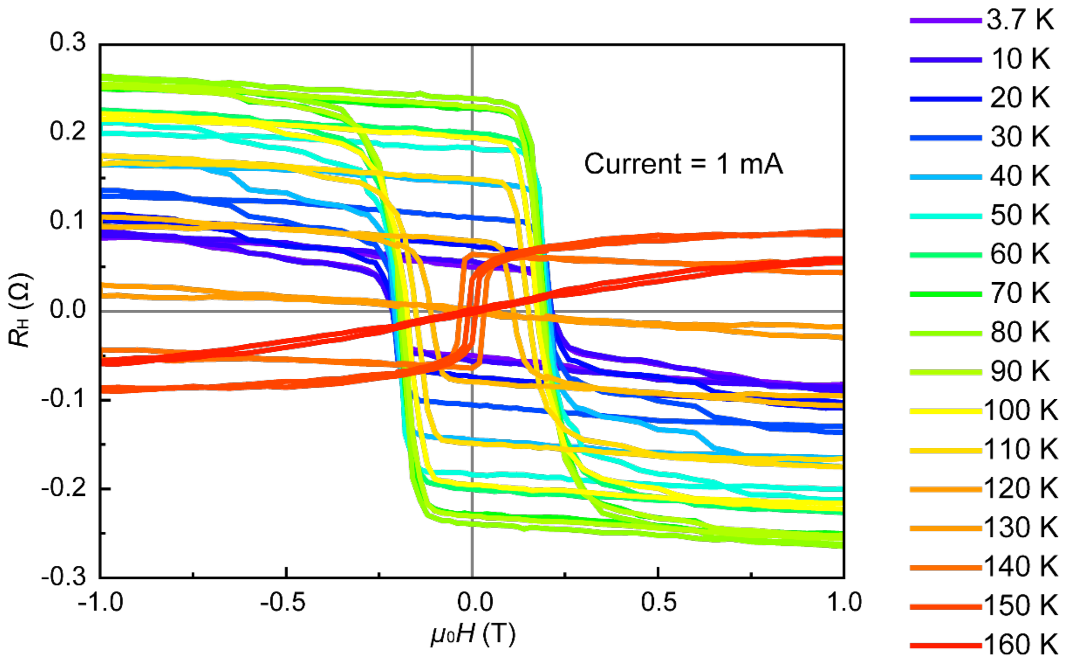

Figure S3. Temperature dependence of the Anomalous Hall effect. The magnetic field is applied along the *z* direction. The Hall resistance R_H is negatively proportional to the perpendicular component of the magnetization in the temperature range from 3.7 K to 120 K.

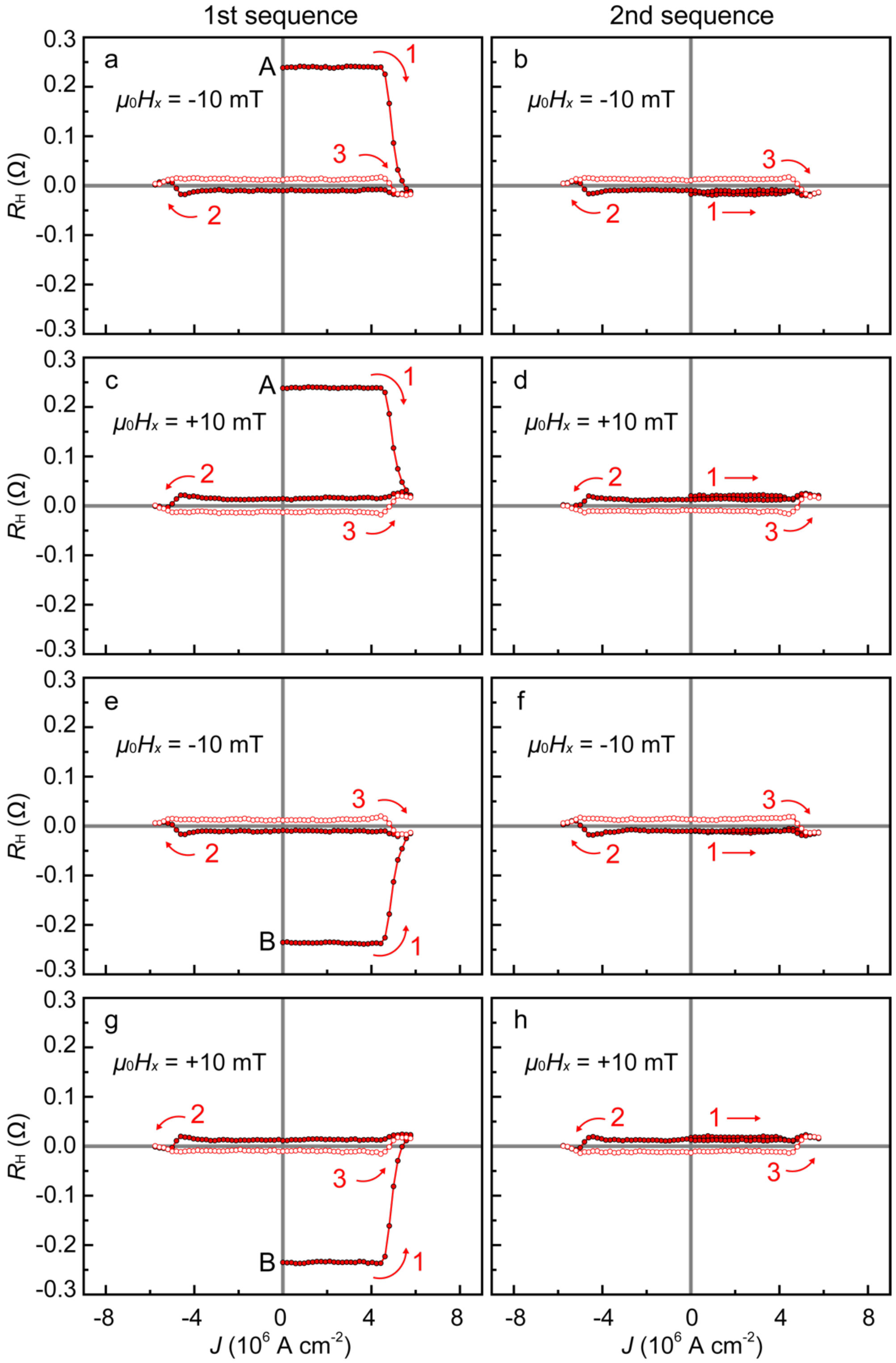

Figure S4. a),c),e),g) R_H–J loops obtained after the initialization process. The values of R_H at A and B correspond to the initial states where the magnetization is aligned along the $-z$ and $+z$ directions, respectively. The measurement process proceeds in the order of 1→2→3. b),d),f),h) R_H–J loops obtained in the second sequence of processes 1, 2, and 3. The above data are taken for a different device that has the same SRO thickness and nearly the same magnetic properties as that shown in the main manuscript. All measurements were carried out at 90 K.

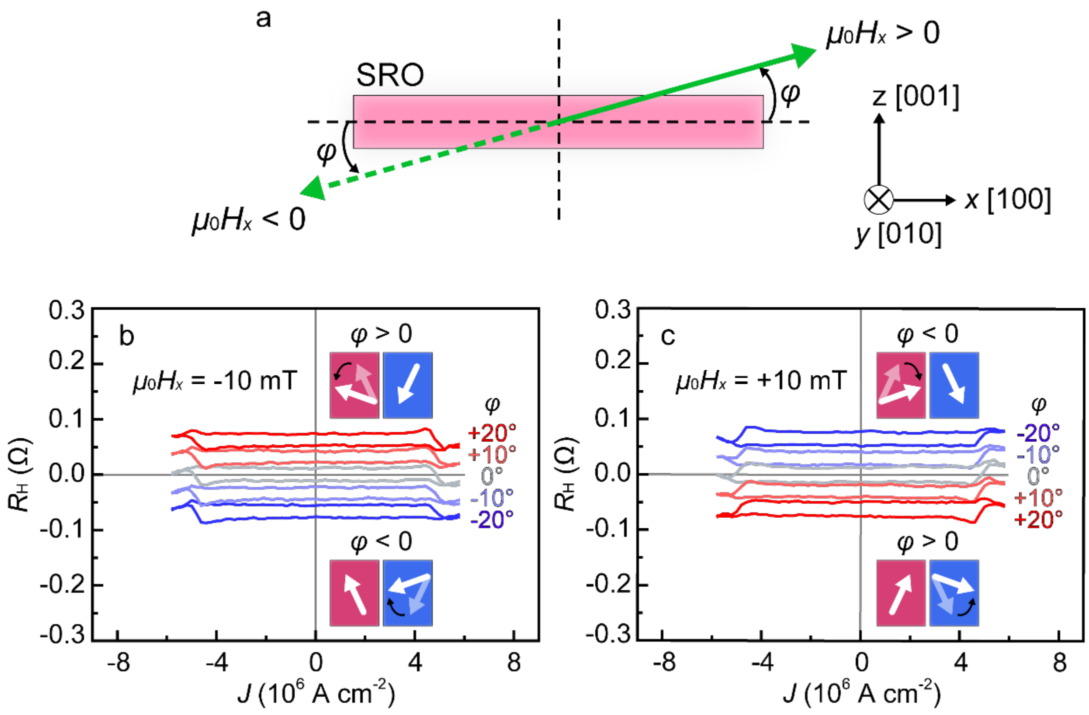

Figure S5. a) Illustration of the misalignment of the sample plane from the H_x direction. The pink rectangle is the side view of the single-layer SRO film. b),c) R_H–J loops obtained at different misalignment angles φ for (b) $\mu_0 H_x = -10$ mT and (c) $\mu_0 H_x = +10$ mT. Insets are the side views of the expected direction of upward (red) and downward (blue) magnetization domains in the mixed region in Figure 5e for $\varphi > 0$ and $\varphi < 0$. The direction of magnetization within each domain transitions from the lighter-colored white arrow to the darker-colored white arrow as $|\varphi|$ increases.

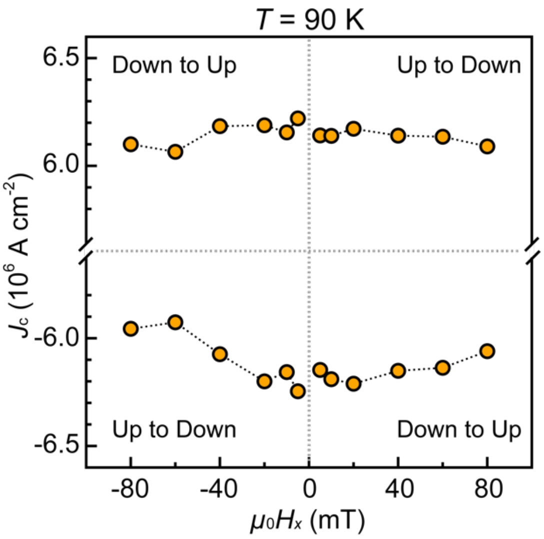

Figure S6. Switching-phase diagram of the relationship between the switching current density J_c and the in-plane field H_x at 90 K. “Up” (“Down”) represents the case where the magnetization points in the $z > 0$ ($z < 0$) direction.

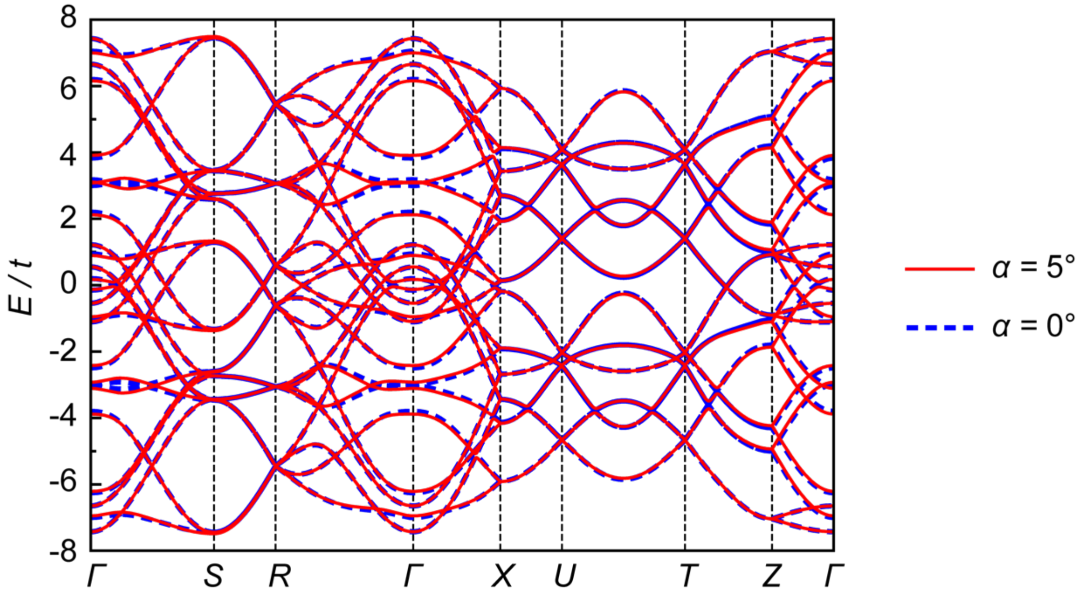

Figure S7. Band structure calculation results. Band structure of SRO for $\alpha = 0°$ and $\alpha = 5°$.

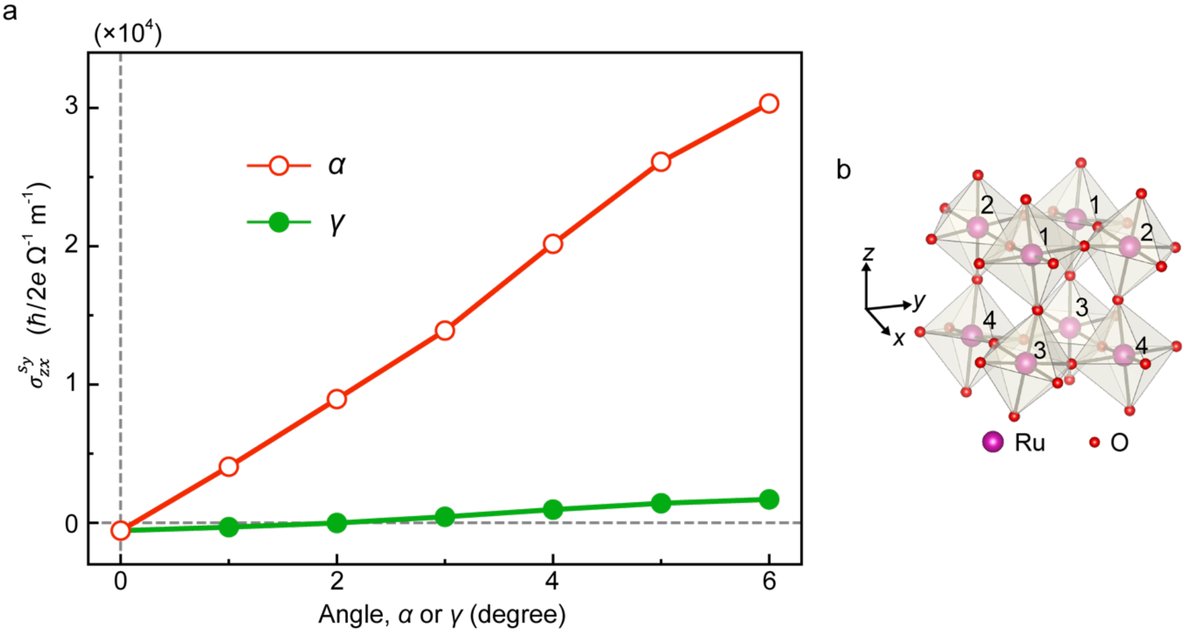

Figure S8. a) Change in $\sigma_{zx}^{s_y}$ of SRO when changing α for $\gamma = 0°$ (red) and when changing γ for $\alpha = 0°$ (green) at $E/t = 0$. b) Illustration of the sublattices of SRO.

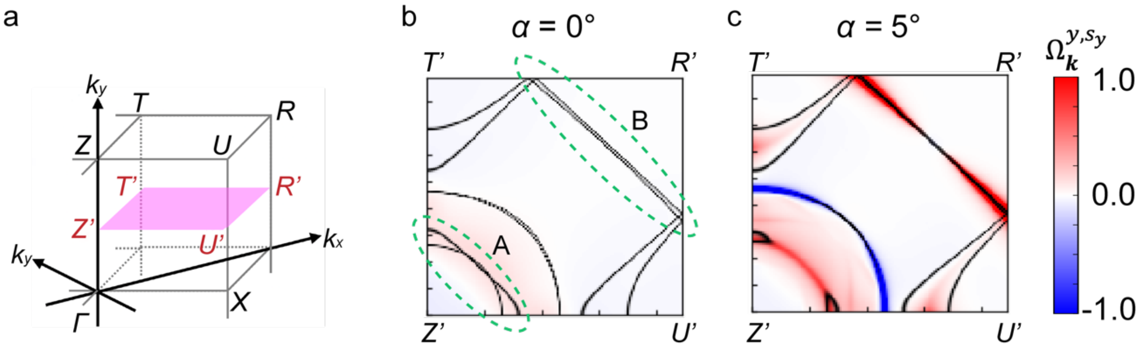

Figure S9. a) Definition of the symmetric points in the $\boldsymbol{k}$-space. b),c) Fermi contours and distribution of the spin Berry curvature $\Omega_{\boldsymbol{k}}^{y,s_y} = \sum_n f(\epsilon_n(\boldsymbol{k}))\Omega_{n\boldsymbol{k}}^{y,s_y}$ in the $\boldsymbol{k}$-space at the energy $E = 0$ for (b) $\alpha = 0°$ and (c) $\alpha = 5°$ calculated from the model. The value of $\Omega_{\boldsymbol{k}}^{y,s_y}$, which is a dimensionless quantity scaled using the lattice parameter (see Section 4), is shown in the range from –1 to +1, expressed as the color scale.

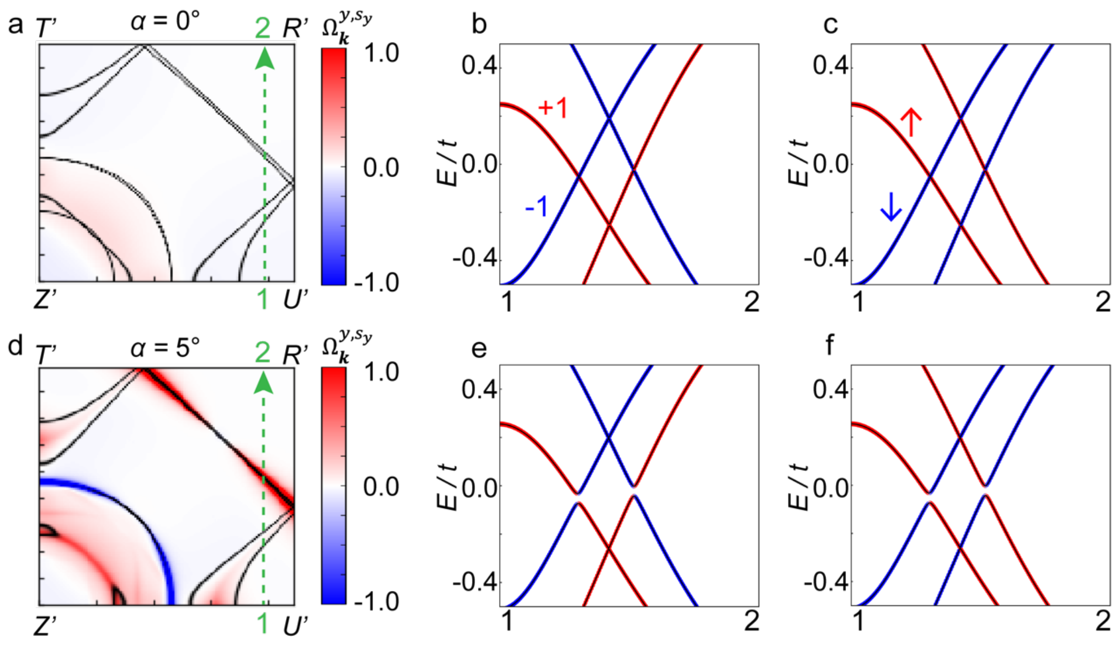

Figure S10. a),d) Fermi contours and distribution of $\Omega_{\boldsymbol{k}}^{y,s_y} = \sum_n f(\epsilon_n(\boldsymbol{k}))\Omega_{n\boldsymbol{k}}^{y,s_y}$ in the $\boldsymbol{k}$-space at the energy $E = 0$ for (a) $\alpha = 0°$ and (d) $\alpha = 5°$ as shown in Figure S9. b),e) Distributions of the parity under sublattice transformation (half-unit cell translation) $T_y^{1/2}$ on each band for (b) $\alpha = 0°$ and (e) $\alpha = 5°$ along the green arrow in (a) and (d), respectively. +1 (red) and –1 (blue) indicates if the Bloch state is even or odd under $T_y^{1/2}$, respectively. c),f) Distributions of the spin polarization s_z for (c) $\alpha = 0°$ and (f) $\alpha = 5°$ along the green arrow in (a) and (d), respectively. ↑ (red) and ↓ (blue) indicate up- and down-spin, respectively.

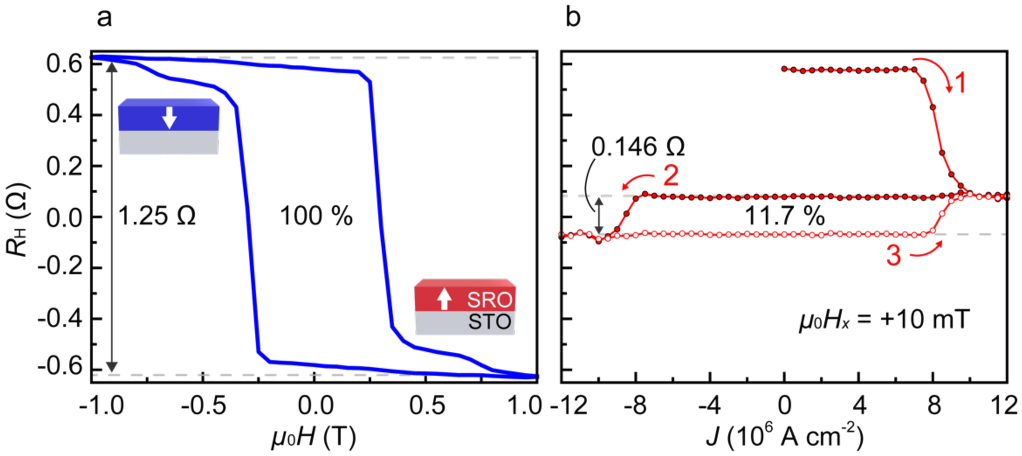

Figure S11. a) AHE loop in the 10-nm-thick SRO film at 90 K, where an external magnetic field $\mu_0 H$ was applied along the z-axis. b) R_H–J loop obtained in the SOT magnetization switching measurement in the same film obtained at 90 K. The white arrows represent the magnetization directions.

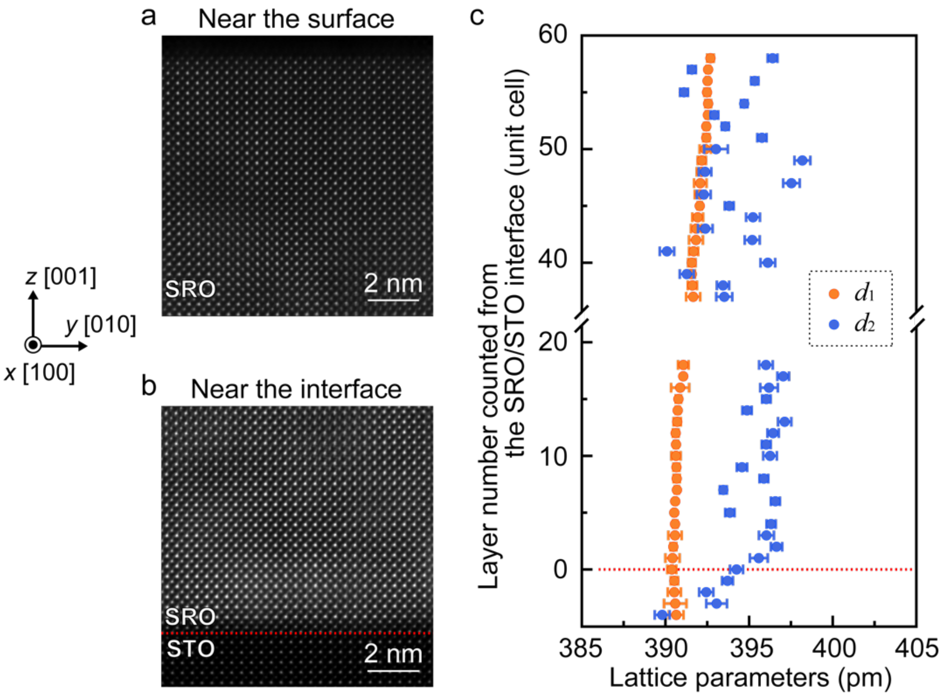

Figure S12. a),b) HAADF-STEM images of a) the surface region (from the 37th to the 58th layers counted from the SRO/STO interface) and b) the interface region (from the −4th to the 18th layers) of the SRO/STO sample obtained at room temperature. The red dashed line denotes the interface of SRO/STO. c) Distribution of the in-plane (d_1) and vertical (d_2) lattice constants along the film-normal direction. Both values were obtained for the Ru (or Ti) atoms. The positive (negative) layer numbers correspond to the RuO_2 (TiO_2) layers within the SRO film (STO substrate).

SI References

[S1] M. Feigenson, J. W. Reiner, L. Klein, *Phys. Rev. Lett.* **2007**, 98, 247204.

[S2] L. Liu, O. J. Lee, T. J. Gudmundsen, D. C. Ralph, R. A. Buhrman, *Phys. Rev. Lett.* **2012**, 109, 096602.

[S3] Y. Chen, D. L. Bergman, A. A. Burkov, *Phys. Rev. B* **2013**, 88, 125110.

[S4] J.-M. Carter, V. Shankar, H.-Y. Kee, *Phys. Rev. B* **2013**, 88, 035111.

[S5] Z. Cui, Y. Zhang, X. Zhai, H. Chen, Y.-D. Chuang, J. Guo, Z. Fu, Z. Li, Y. Lu, *Phys. Rev. B* **2022**, 106, 024424.

[S6] O. J. Lee, L. Q. Liu, C. F. Pai, Y. Li, H. W. Tseng, P. G. Gowtham, J. P. Park, D. C. Ralph, R. A. Buhrman, *Phys. Rev. B* **2014**, 89, 024418.

[S7] I. Shin, W. J. Cho, E.-S. An, S. Park, H.-W. Jeong, S. Jang, W. J. Baek, S. Y. Park, D.-H. Yang, J. H. Seo, G.-Y. Kim, M. N. Ali, S.-Y. Choi, H.-W. Lee, J. S. Kim, S. D. Kim, G.-H. Lee, *Adv. Mater.* **2022**, 34, 2101730.

[S8] Y. Ou, Z. Wang, C. S. Chang, H. P. Nair, H. Paik, N. Reynolds, D. C. Ralph, D. A. Muller, D. G. Schlom, R. A. Buhrman, *Nano Lett.* **2019**, 19, 3663–3670.

[S9] Q. Xie, W. Lin, J. Liang, H. Zhou, M. Waqar, M. Lin, S. L. Teo, H. Chen, X. Lu, X. Shu, L. Liu, S. Chen, C. Zhou, J. Chai, P. Yang, K. P. Loh, J. Wang, W. Jiang, A. Manchon, H. Yang, J. Chen, *Adv. Mater.* **2022**, 34, 2109449.

[S10] L. Liu, Q. Qin, W. Lin, C. Li, Q. Xie, S. He, X. Shu, C. Zhou, Z. Lim, J. Yu, W. Lu, M. Li, X. Yan, S. J. Pennycook, J. Chen, *Nat. Nanotechnol.* **2019**, 14, 939−944.

[S11] D. Kim, H. Lim, S. S. Ha, O. Seo, S. S. Lee, J. Kim, K. Kim, L. P. Ramirez, J.-J. Gallet, F. Bournel, J. Y. Jo, S. Nemsak, D. Y. Noh, B. S. Mun, *J. Chem. Phys.* **2020**, 152, 034704.

[S12] S. H. Chang, Y. J. Chang, S. Y. Jamg, D. W. Jeong, C. U. Jung, Y.-J. Kim, J.-S. Chung, T. W. Noh, *Phys. Rev. B* **2011**, 84, 104101.

[S13] K. A. Müller, W. Berlinger, F. Waldner, *Phys. Rev. Lett.* **1968**, 21, 814.

[S14] R. Loetzsch, A. Lübcke, I. Uschmann, E. Förster, V. Große, M. Thuerk, T. Koettig, F. Schmidl, P. Seidel, *Appl. Phys. Lett.* **2010**, 96, 071901.
